# A meta-analysis of the traditional herb Zicao and its active components for atopic dermatitis

**DOI:** 10.3389/fphar.2025.1648894

**Published:** 2026-01-06

**Authors:** Qin-yao Wu, Ya-yi Jiang, Jun-e Ming, Ping-sheng Hao

**Affiliations:** Hospital of Chengdu University of Traditional Chinese Medicine, Chengdu, China

**Keywords:** animal model, *Arnebia euchroma*, atopic dermatitis, effect, mechanism, *Lithospermum erythrorhizon*

## Abstract

**Background:**

The traditional Chinese herb Zicao and its bioactive constituents demonstrate therapeutic potential for atopic dermatitis (AD), and a systematic review assessing its effectiveness for managing AD is still lacking.

**Purpose:**

This meta-analysis aimed to synthesize the effects of Zicao on AD animal models and elucidate the underlying mechanisms.

**Methods:**

Ten databases (PubMed, Embase, Cochrane, Web of Science, China National Knowledge Internet (CNKI), VIP, CBM, Wanfang, Google Scholar, and ProQuest Dissertations & Theses Global) were systematically searched from the inception through May 2025. Study quality was assessed using SYRCLE’s risk-of-bias tool. Random-effects models pooled standardized mean differences (SMDs) with 95% confidence intervals (CIs) for primary outcomes (dermatitis severity and scratching behavior) and secondary outcomes (cytokines, epidermal thickness, and filaggrin (FLG)). Subgroup analyses examined the animal species, modeling method, drug formulation, and intervention protocol. Publication bias was evaluated via funnel plots and Egger’s regression; sensitivity analyses utilized the leave-one-out methodology. Analyses were conducted in R version 4.3.2 software.

**Results:**

Ten studies (n = 316 animals) revealed the following: Zicao treatment significantly decreased the severity of dermatitis (SMD = −3.30, 95% CI: −4.37 to −2.23; *p* < 0.001) and scratching behavior (SMD = −2.60, 95% CI: −3.76 to −1.44; *p* < 0.01). In addition, Zicao treatment significantly decreased cytokines: TNF-α, thymic stromal lymphopoietin (TSLP), IL-4, IL-13, IgE, and mast cell infiltration, whereas no significant effects were observed for IFN-γ, IL-6, epidermal thickness, or FLG expression.

**Conclusion:**

The traditional Chinese herb Zicao ameliorates AD symptoms and Th2-associated inflammation but exhibits limited efficacy in epidermal barrier restoration. However, the pooled effect estimates from this meta-analysis must be interpreted with caution as the preliminary indications of potential efficacy rather than as conclusive evidence, given the widespread methodological limitations and the absence of pharmacokinetic and toxicological data in the included studies. Therefore, future investigations using chemically standardized preparations and comprehensive safety assessments are needed to validate these findings.

**Systematic Review Registration:**

https://www.crd.york.ac.uk/PROSPERO/, identifier CRD42023449172.

## 1 Introduction

Atopic dermatitis (AD) is a prevalent, chronic inflammatory skin disorder affecting substantial pediatric and adult populations globally, and it is characterized by intense pruritus and eczematous lesions. The disease significantly impairs the quality of life, including psychological comorbidities, and one study showed that its prevalence is steadily increasing worldwide (GBD, 2021 Asthma and Allergic Diseases Collaborators, 2025; Sandhu et al., 2019). Topical corticosteroids and calcineurin inhibitors are frequently used as first-line treatments, and for moderate to severe cases, biologics and targeted therapies are considered advanced treatment options. However, concerns regarding the side effects, variable efficacy, and cost underscore the need for continued exploration of alternative therapeutic agents (Lin et al., 2025; Hagenström et al., 2024; Broeders et al., 2016).

The root of *Lithospermum erythrorhizon* Siebold & Zucc., *Arnebia euchroma* (Royle) Johnst., or *Arnebia guttata* Bunge (all belonging to the Boraginaceae family), known as Zicao, has been used in traditional Chinese medicine systems for centuries to treat inflammatory skin conditions such as burns, wounds, and eczema (Li-Na et al., 2025). The primary bioactive constituents of Zicao are naphthoquinone compounds, including shikonin, acetylshikonin, and β,β-dimethylacrylshikonin. Although *Lithospermum erythrorhizon* (LE) was removed from the Pharmacopoeia of the People’s Republic of China after 2015, historical studies on traditional Chinese medicinal plants confirm that it was indeed the botanical source of Zicao in ancient formulations. Its removal likely reflects concerns over the scarcity of domestic resources and challenges in commercial production (Li-Na et al., 2025; Jia-Xin et al., 2018). In addition, the medicinal plant databases of other countries, such as Japan’s NIHS Research Center for Medicinal Plant Resources and Korea’s TM-MC 2.0, continue to recognize LE as a legitimate source of Zicao (Korea Institute of Oriental Medicine, 2014; Research Center for Medicinal Plant Resources, 2025).

Some clinical studies have demonstrated the therapeutic potential of Zicao in AD, with topical shikonin-containing ointments reducing Eczema Area and Severity Index (EASI) scores (Yen and Hsieh, 2016) and oral LE extract improving stratum corneum hydration and ceramide levels in patients with AD (Cho et al., 2008). Moreover, compound Zicao oil preparations have demonstrated efficacy akin to pimecrolimus, accompanied by favorable safety profiles (Zhang et al., 2019a).

Preclinical evidence indicates that Zicao inhibits the type-2 inflammatory cascade by inhibiting essential mediators such as thymic stromal lymphopoietin (TSLP), IL-4, and IL-13 (Le et al., 2022). Shikonin, the principal bioactive constituent, exerts anti-inflammatory, antimicrobial, and antioxidant activities (Sun et al., 2022). This multi-target mechanistic profile positions Zicao and its constituents as promising candidates for AD intervention. However, the preclinical therapeutic efficacy and associated outcome metrics of Zicao in AD have not been systematically evaluated. Therefore, this meta-analysis aims to critically appraise and synthesize the existing preclinical evidence base to comprehensively assess the therapeutic potential of Zicao in AD.

## 2 Materials and methods

This meta-analysis was carried out according to the protocol proposal published in PROSPERO. The entire process was implemented in accordance with PRISMA requirements (Moher et al., 2009), and the registration code for this study protocol is CRD42023449172 (the specific address is PROSPERO).

### 2.1 Search strategy and study selection

A systematic search was conducted across PubMed, Embase, Cochrane, WOS, China National Knowledge Internet (CNKI), VIP Information Chinese Periodical Service Platform (VIP), China Biology Medicine disc (CBM disc), and Wanfang Data Knowledge Service Platform (Wanfang) databases. The search period encompassed the whole duration of each database, starting from its establishment and concluding in May 2025. The primary objective of the investigation centered on studying the effect of LE and its active components on animals suffering from atopic dermatitis using a Boolean search strategy with the operators “AND,” “OR,” and “NOT”: atopic dermatitis, atopic neurodermatitides, Lithospermum, Radix Arnebiae, Zicao, shikonin, and acetylshikonin (the search syntax can be found in Supplementary Material). This search was conducted without any limitations on the language, country, or publication.

To mitigate potential publication bias and the effects of access restrictions due to institutional journal subscriptions, the following supplementary strategies were implemented: searching Google Scholar and ProQuest Dissertations & Theses Global for unpublished studies, conference abstracts, and academic theses that could have useful information. We used several methods to obtain the full articles for records that were chosen for full-text review but were not available through our institutional subscriptions. These methods included direct requests to the authors, searches on academic social networks such as ResearchGate, and interlibrary loan services.

### 2.2 Inclusion criteria

This meta-analysis aimed to evaluate the efficacy of interventions derived from Zicao or its primary bioactive constituents (e.g., shikonin) in animal models of atopic dermatitis. The review included randomized controlled animal trials that reported on symptomatic improvement and associated mechanisms: (1) AD animal models, without any limitations on gender, species, or method of modeling; (2) treatment groups using Zicao (defined as the root of *Lithospermum erythrorhizon* Sieb. and Zucc., *Arnebia euchroma* (Royle) Johnst., or *Arnebia guttata* Bunge) in the form of crude extracts, its purified chemical derivatives (e.g., shikonin and acetylshikonin), or compound preparations in which Zicao serves as the principal component. There were no restrictions on the dosage, formulation, route of administration, or treatment duration. (3) The control group received either the vehicle alone (vehicle control) or were left untreated (untreated control). (4) The primary outcome included dermatitis severity (e.g., SCORing Atopic Dermatitis (SCORAD) and Eczema Area and Severity Index), scratching behavior (including scratch frequency, scratch duration, and scratching intensity). (5) The secondary outcome included histological alterations in the skin, immune-associated cytokines, and other relevant outcome measures. The exclusion criteria were as follows: (1) studies involving non-mammalian species, *in vitro* experiments, animal models of comorbidities, human studies, and in silico studies; (2) reviews, case reports, patents, and commentaries; (3) without a control group or only including before-and-after controlled studies in the treatment group; (4) studies where LE and its derivatives were not the main intervention; and (5) no reporting of the outcome.

### 2.3 Data extraction

These works were independently examined by two writers using the pre-established inclusion and exclusion criteria. The process started with a brief review of the titles and abstracts, followed by an in-depth examination of the entire text in order to evaluate its content. Any disagreements during screening were resolved by consensus. The extracted data were then recorded in a standardized Excel spreadsheet. The extracted information included (1) the title; (2) the first author and the publication date; (3) animals (species, sex, number, age, and weight); (4) the approach of disease modeling; (5) medication administered to the experimental group, including the name of the medication, the dose, and the dosing frequency; (6) the non-functional substances in the control group (the name, dose, and dosing frequency); (7) the intervention and the duration; and (8) the outcome: the data for the outcome were expressed as the mean ± SD. For data presented only in graphical form, numerical values were digitized using GetData Graph Digitizer (version 2.26.0.20), and to ensure accuracy, all digitized values were independently extracted by two reviewers. If conflicts arose, a third reviewer was involved in resolving them. For studies featuring multiple intervention groups with varying doses, the outcomes from these groups were pooled to create a single, combined estimate. This procedure, endorsed by the Cochrane Handbook, prevents unit-of-analysis errors by ensuring that no participants are double-counted in the meta-analysis (Cochrane, 2004). If the data presented in the text were initially expressed as standard errors (SEs), they were afterward transformed into standard deviations (SDs) (Lee et al., 2015).

Additionally, all medicinal plant species mentioned in this systematic review were taxonomically validated to ensure nomenclatural accuracy. The accepted scientific name, author citation, and family for each species were verified using the Plants of the World Online (POWO) database and the Medicinal Plant Names Services (MPNS) portal. The standard pharmacopeial drug name (where applicable) is also provided.

### 2.4 Risk-of-bias and quality assessment

The Systematic Review Center for Laboratory Animal Experimentation (SYRCLE) risk-of-bias tool was used for bias assessment (Hooijmans et al., 2014). It assesses bias in the experimental design, execution, measurement, reporting, and other sources, each of which is categorized into three risk-of-bias levels: “Yes,” meaning low risk, “No,” meaning high risk, and “Unclear,” meaning unclear. The assessment of the reporting quality of the included studies was performed using a scoring system adapted from Liu et al. (2024), where each domain received a score of 1 = Yes, 0 = No, 0.5 = partly, and NA = not applicable, contributing to a total score of 10 points. Two authors assessed the studies independently. Any disagreements were resolved by consultation with a third reviewer.

### 2.5 Statistical analysis

Statistical analysis was performed using R version 4.3.2 software (the {meta} package). The outcome indicators were represented as continuous variables; the combined total effect sizes were presented using the standardized mean difference (SMD) and 95% confidence intervals (95% CI). A *p*-value < 0.05 indicates statistical significance. Consolidation of subgroup effect sizes via Hedges’ g—in accordance with Cochrane Collaboration guidelines—reduced bias attributable to the duplicate use of shared control group data in SMD calculations. A random-effects model was used since the actual effect size varied across studies because of diverse animal and treatment factors (Muka et al., 2020). Confidence intervals for the pooled effect estimates were computed using Knapp–Hartung adjustments (Harrer et al., n.d.).

Heterogeneity was quantitatively assessed through three complementary metrics: the percentage of variability in the effect sizes not attributable to sampling error (I^2^), the variance of the distribution of true effect sizes (Tau^2^/τ^2^), and the prediction intervals (PIs) (Harrer et al., n.d.; IntHout et al., 2016). The magnitude of heterogeneity was quantified by (1) Cochrane Handbook thresholds: I^2^ = 25% (low), 50% (moderate), and 75% (substantial); and (2)τ^2^ (the DerSimonian–Laird method estimator), which represents the variance of the true effect sizes across studies, quantifying the absolute magnitude of between-study heterogeneity. τ^2^ = 0 indicates the absence of between-study heterogeneity (all observed variability is attributable to sampling error alone). τ^2^ > 0 signifies the presence of between-study heterogeneity, with larger values corresponding to greater heterogeneity magnitude. (3) Computation of the 95% prediction interval bounds for the true effect size. Using the prediction interval delineates the expected range of effects in future studies based on current evidence. Crucially, when the interval lies entirely on the intervention-beneficial side of the null line, it predicts consistent therapeutic benefit despite heterogeneity. Conversely, intervals spanning the null value (e.g., risk ratio = 1 or mean difference = 0) indicate uncertain clinical relevance.

Subgroup analyses were restricted exclusively to the primary outcome measures. Systematic investigation of outcome variation across experimental settings and sources of heterogeneity was performed using predetermined subgroup criteria, including, but not limited to, animal species, modeling method, drug formulation, drug source, and intervention protocol. We used the Influence Analysis function from the {dmeta} package (R) to perform the sensitivity analysis and assess the presence of outliers, the former using the leave-one-out method to examine the effect of each finding on the effect size and I^2^ one-by-one effect (Viechtbauer and Cheung, 2010). Publication bias assessment for the primary outcomes utilized dual analytical approaches: visual inspection of funnel plot symmetry and the quantitative Egger’s linear regression test.

## 3 Results

### 3.1 Study selection

**FIGURE 1:**
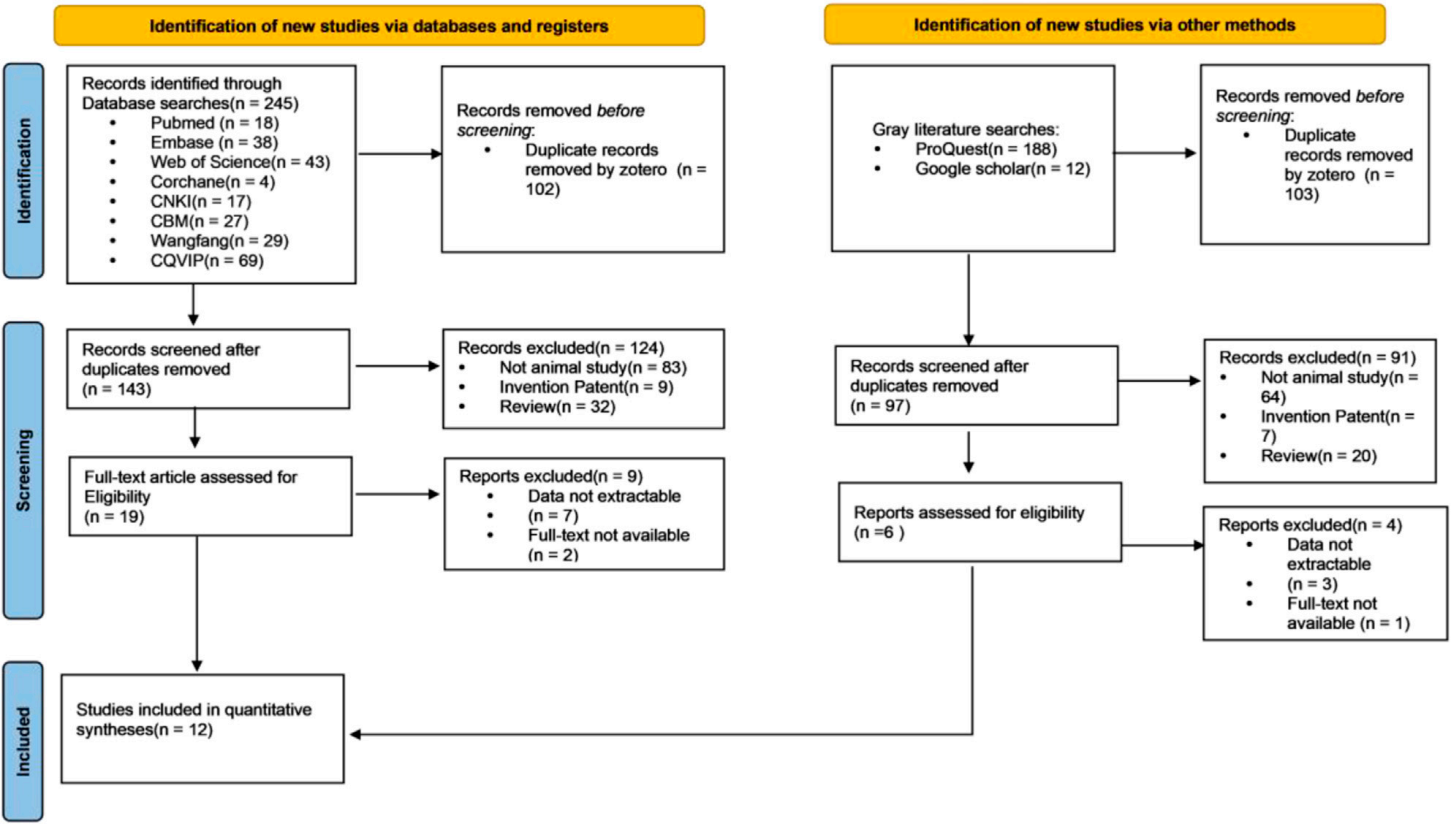
PRISMA 2020.

A thorough search across eight databases found a total of 445 relevant studies. Among them, 18 papers were obtained from PubMed, 38 papers were from Embase, 4 papers were from Cochrane, 43 papers were from Web of Science (WOS), 29 papers were from Wanfang, 17 papers were from CNKI, 69 papers were from CQVIP, 27 papers were from China Biology Medicine (CBM), 188 papers were from ProQuest, and 12 papers were from Google Scholar. Following relevant exclusions, a total of 12 studies ultimately met all the inclusion criteria. The literature selection process is shown in Figure 1.

### 3.2 Characteristics of included studies

Following systematic screening, 12 studies met the final inclusion criteria (Oh et al., 2021; Kadoyama et al., 2019; Ku et al., 2018; Choi et al., 2017; Lee et al., 2009; Kim et al., 2009; Wu et al., 2021; Xiaochao et al., 2019; Liyuan et al., 2019b; Xu et al., 2019), comprising four Chinese and six English publications. The experimental models utilized NC/Nga mice (k = 4), BALB/c mice (k = 4), KM mice (k = 1), SD rats (k = 2), and unspecified murine species (k = 1); the detailed animal characteristics (sex/age/weight) are tabulated in Table 1. The majority of studies (k = 8) employed dinitrochlorobenzene (DNCB) sensitization to induce dermatitis. Other induction methods comprised ovalbumin (OVA) challenge (k = 1), oxazolone (OX) exposure (k = 1), a spontaneous model (k = 1), and the combined use of Biostir AD ointment with 4% SDS (k = 1). The therapeutic interventions involved commercial Chinese polyherbal preparation (k = 6), shikonin (k = 2), an investigator-formulated botanical preparation (k = 1), and standardized extracts (k = 3), which were administered via oral gavage (k = 4) or external application (k = 8). Furthermore, none of the included studies reported any toxicological or safety assessments for the administered preparations.

**TABLE 1:** Characteristics of included studies.

Author, year	Species (sex,age,n = treatment/model group, weight)	Modeling method	Intervention Drug	Source/Botanical Source	Plant Part Used	Extraction and Preparation Method	Chemical Characterization/Standardization	Intervention (administration, dosage, duration)
Oh2021	NC/Nga mice (male,4 weeks,6/6,NA)	DNCB; 14 weeks	Dried roots of Lithospermum erythrorhizon (LR) extract	Lithospermum erythrorhizon Siebold & Zucc. [Boraginaceae]	Root	Reflux extraction with 70% ethanol (1:3, w/v) at 85 °C–90 °C, 2 h × 2. Combined extracts were filtered and lyophilized	0.23% lithospermic acid/HPLC analysis at 320 nm	By Intragastric; (50, 100, and 200)mg/kg; daily; 4°weeks
zhangli2019	BALB/c mice (male,6–8 weeks,8/8,20 ± 2 g)	DNCB; 8 weeks	Fufang Zicao You^a^	Zicao (Not specified)	Root	Not reported	Not reported	By external; 200 μL,twice/d; 1°week
Wu2021	BALB/c mice (male,6–8 weeks,15/15,15–20 g)	DNCB; 25 days	(−)-Shikonin	Chemical synthesis: (Supplier: BioPush, PS2952-0025)	Not reported	Not reported	Purity ≥98% (HPLC)	By Intragastric; (20,30,and 40)mg/kg; daily 15°days
Yang2019	SD rat (male/female,NA,8/8,180–220 g)	DNCB; 4 weeks	Zicao oil^b^	Zicao (Not specified)	Not reported	Not reported	Not reported	By external; NA,twice/d; 12 days
Zhang2019	Kunming (KM) mice (male/female,4–5 weeks,10/10,18–22 g)	OVA; 4 weeks	ZiCao YingEr RuanGao^c^	Zicao (Not specified)	Bark	Not reported	Not reported	By external; (0.5,1,and 2)g/kg,twice/d; 14 days
Lee JH2009	NA mice (female,7 weeks,7–8/7–8,21.19 ± 1.74 g)	Oxazolone; 20 days	Dried roots of Lithospermum erythrorhizon (LR) extract	Lithospermum erythrorhizon Siebold & Zucc. [Boraginaceae]	Root	Reflux extraction with 70% ethanol (1:3, w/v) at 85 °C–90 °C, 2 h × 2. Combined extracts were filtered and lyophilized	0.23% lithospermic acid/Not reported	By Intragastric; (100, 250, and 500)mg/kg/d; 20°days
Kim J2009	NC/Nga mice (male,6 weeks,10/10,NA)	Spontaneous Induction; 10 weeks	Dried roots of Lithospermum erythrorhizon (LR) extract	Lithospermum erythrorhizon Siebold & Zucc. [Boraginaceae]	Root	Reflux extraction with 70% ethanol (1:5, w/v) at 85 °C–90 °C, 12 h × 2. Combined extracts were filtered and lyophilized	2.0 mg/g lithospermic acid/Not reported	By Intragastric; NA; 10°weeks
Ku JM2018	BALB/c mice (male,6 weeks,8/8,NA)	DNCB; 11 days	Jawoongo ointment^d^	Lithospermum erythrorhizon Siebold & Zucc. [Boraginaceae]	Root	Not reported	Not reported	By external; NA; 2°weeks
Jeong-Hae Choi2017	NC/Nga mice (male,6 weeks,5/5.25 g)	DNCB; 7 weeks	Jaungo^e^	Lithospermum erythrorhizon Siebold & Zucc. [Boraginaceae]	Root	Not reported	Not reported	By external; 200 mg; daily; 3°weeks
Kadoyama K2019	NC/Nga mice (male,10 weeks,15/15,NA)	Biostir AD; 3 weeks	Shikonin	Chemical synthesis: (Supplier: Wako pure chemical industries, 191–13331)	Not reported	Not reported	Purity ≥99% (HPLC)	By external; 10 μmol/L,daily; 20°days
Ma2022	SD rat (NA,5–7 weeks, 10/10,180–220 g)	DNCB; 37 days	Compound Turkish gall ointment (CTGO)^f^	Arnebia euchroma (Royle ex benth.) I.M.Johnst. [Boraginaceae]	Root	Not reported	Untargeted UHPLC-Q-Orbitrap MS(No quantitative data were reported)	By external; (0.035,0.07,and 0.14)g/g,4 h/daily; 14°days
Hu2025	BALB/c mice (male/male,6–8weeks,6/6,18–20 g)	DNCB; 26 days	Lithospermum erythrorhizon oil	Lithospermum erythrorhizon Siebold & Zucc. [Boraginaceae]	Root	Lithospermum erythrorhizon roots were macerated in olive oil (1:5, w/v) at 40 °C for 72 h	Not reported	By external; 200 μL, twice/d; 14°days

^a^ Other botanical drug(s) included: *Lonicera japonica* Thunb[Caprifoliaceae], Angelica dahurica (Hoffm.) Benth. and Hook.f. ex Franch. and Sav[Apiaceae],and (+)-BORNEOL.

^b^ Other botanical drug(s) included: the oil of Olea europaea L[Oleaceae].

^c^ Other botanical drug(s) included: Carthamus tinctorius L[Asteraceae].

^d^ Other botanical drug(s) included: Angelica sinensis (Oliv.) Diels[Apiaceae].

^e^ Other botanical drug(s) included: Angelica sinensis (Oliv.) Diels[Apiaceae].

^f^ CTGO: the gall of Quercus infectoria G.Olivier[Fagaceae] and the dried root of Arnebia euchroma(Royle ex Benth.) I.M.Johnst[Boraginaceae].

### 3.3 Risk of bias and quality of the included studies

**FIGURE 2:**
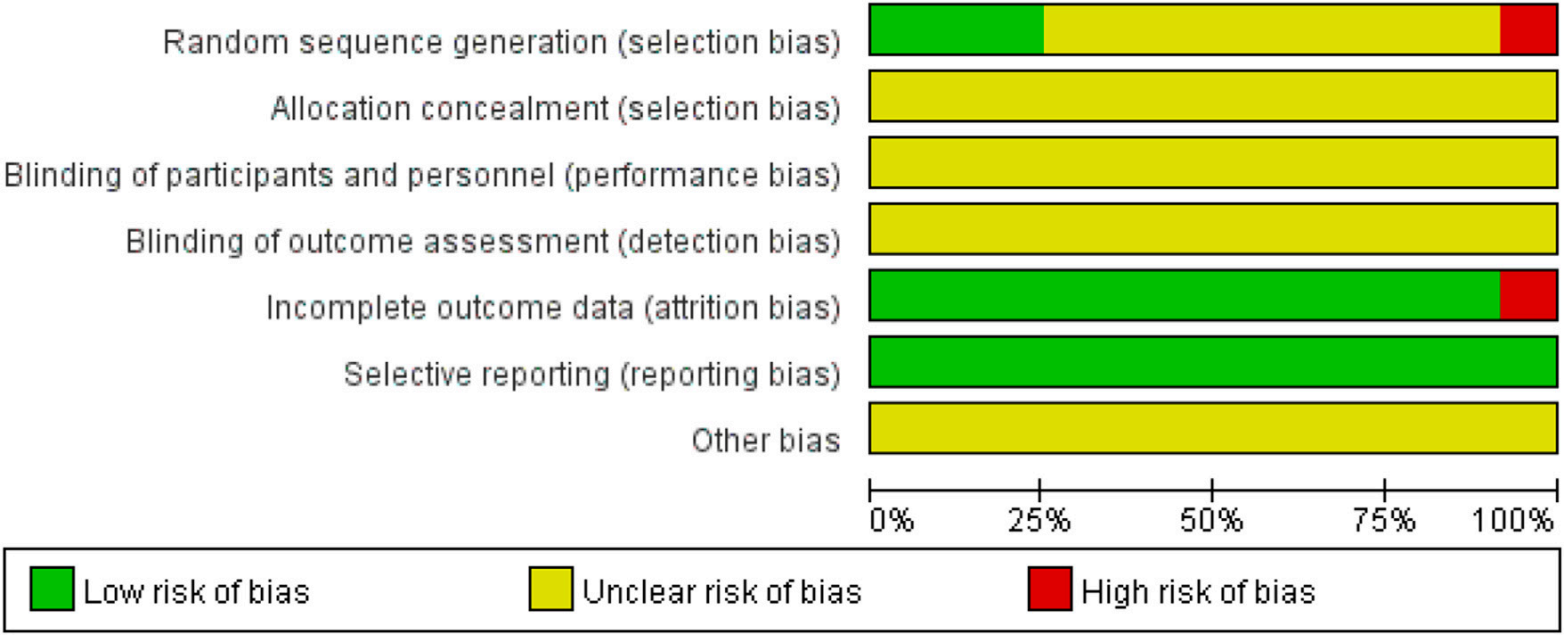
Risk-of-bias graph.

The risk-of-bias assessment for all 12 included studies (summarized in Figure 2) revealed critical methodological limitations: (1) allocation methodology was unreported in one study (Lee et al., 2009), while only three of the remaining nine studies detailed random sequence generation procedures (Wu et al., 2021; Liyuan et al., 2019b; Xu et al., 2019); (2) allocation concealment was universally unreported; (3) all studies failed to report the blinding of investigators, randomization implementation, or outcome assessor masking; (4) one study reported the death of animals during disease modeling after randomization but did not provide details regarding the missing data from dead animals and the potential impact on the outcomes (Liyuan et al., 2019b); and (5) one included study (Lee et al., 2009) reported sample sizes with uncertainty, indicating ranges of n=(7–8) for both the experimental and control groups. In accordance with the principle of conservative estimation when handling imprecise numerical data in meta-analysis, we adopted the lower bound of the reported range (n = 7) for effect size calculations. This approach minimizes the risk of overestimating the precision in pooled effect estimates as using smaller sample sizes yields wider confidence intervals and appropriately reflects the underlying uncertainty in the primary data. (6) As detailed in the table of the included study characteristics (Table 1), all studies were assessed as having a potential risk of bias.

**FIGURE 3:**
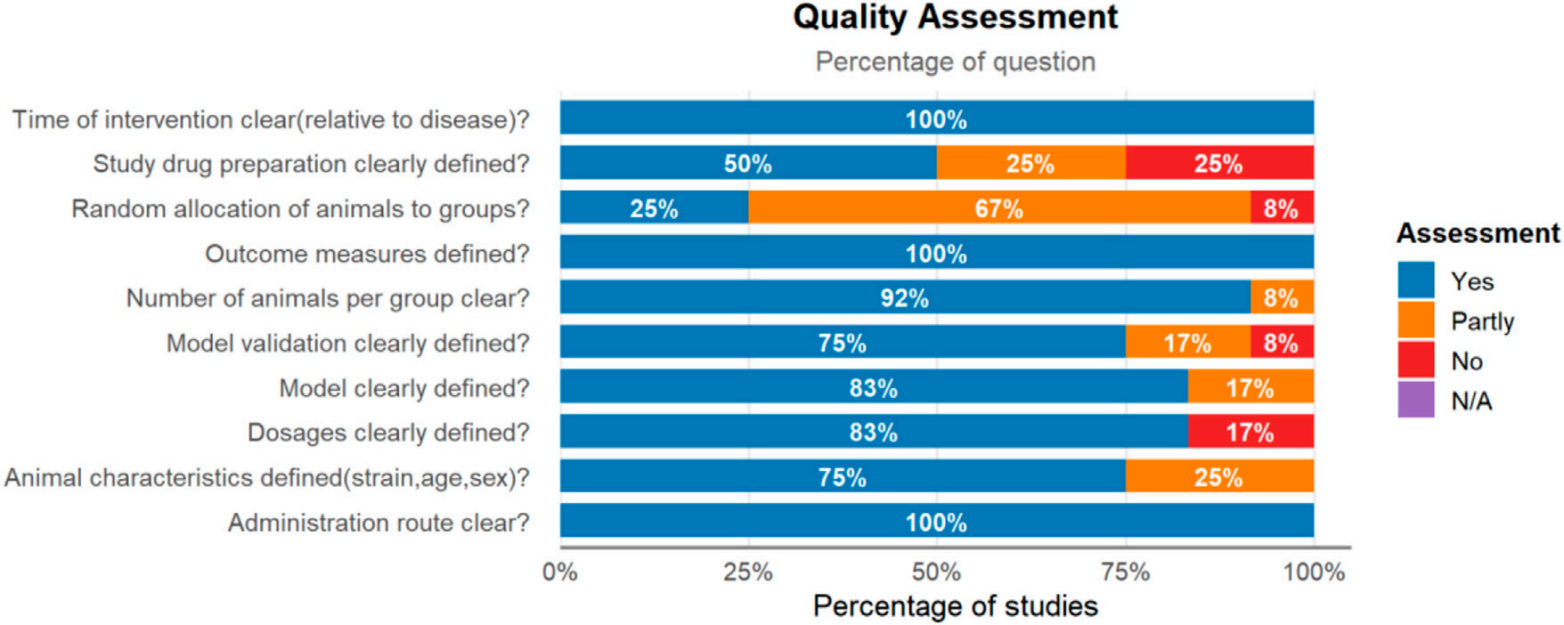
Quality assessment.

The reporting quality was assessed with a 10-item checklist, with scores ranging from 3 to 10 (Figure 3). Among 12 studies, one study received a score of 10 (Liyuan et al., 2019b). All studies demonstrated high quality, with 78.3% of the parameters receiving a “yes” response. Nine studies (75%) clearly defined animal characteristics (species, age, sex, and weight). A total of 11 studies (91.6%) clearly reported the number of animals per group at the start of the experiment. All studies clearly defined the disease model, with nine studies (75%) providing validation of the model. Six studies (50%) clearly defined the study drug preparation method, 10 studies (83.3%) clearly defined the dosages, and all studies defined the administration route and treatment duration. While the reporting of methodological procedures such as randomization was limited (reported in 25% of studies), all included studies provided well-defined outcome measures and complete datasets, allowing for their inclusion in the quantitative synthesis.

### 3.4 Primary outcomes

#### 3.4.1 Dermatitis severity: SCORAD and EASI

##### 3.4.1.1 Effect sizes and heterogeneity

In nine included studies, the dermatitis severity score was used as a quantitative measure to assess the manifestations of AD (Oh et al., 2021; Kadoyama et al., 2019; Choi et al., 2017; Wu et al., 2021; Xiaochao et al., 2019; Liyuan et al., 2019b; Xu et al., 2019; Hu et al., 2025; Ma et al., 2022). Heterogeneous assessment tools were utilized, including the EASI (Xu et al., 2019; Liyuan et al., 2019a) and SCORAD (Wu et al., 2021; Liyuan et al., 2019b; Xu et al., 2019), with Liyuan et al. (2019b) reporting exclusively per-item scores. The severity of dermatitis symptoms (e.g., erythema, hypertrophy, and scaling) was assessed using standardized scoring criteria (typically on a scale of 0 -3). The composite score derived from these criteria positively correlated with the overall severity of AD. Therefore, in accordance with the Cochrane Handbook, pooled SMDs were calculated using a random-effects model. To compute the composite standard deviation for Liyuan et al. (2019b), the variance summation principle was applied, given the statistical independence of the variables—where the variance of a sum equals the sum of variances (Macro, 2012)—followed by a square-root transformation to revert to the standard deviation units.

**FIGURE 4:**
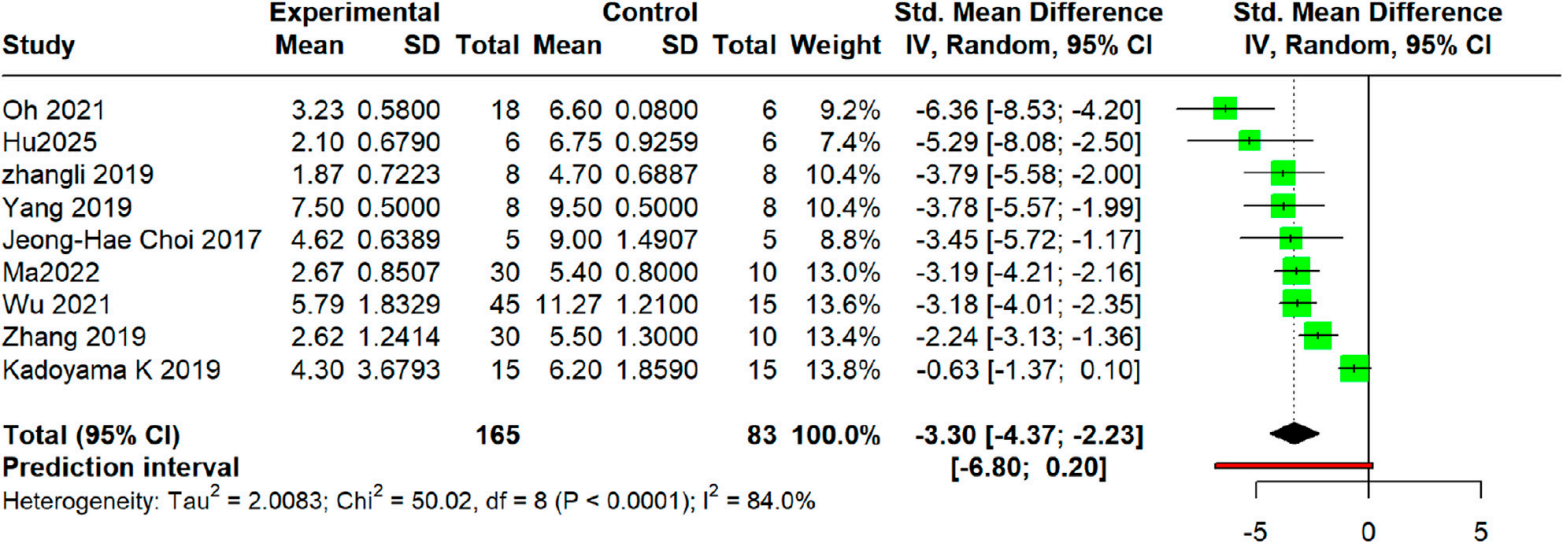
Meta-analysis of the severity of Dermatitis.

Meta-analysis of nine studies demonstrated that the intervention significantly reduced dermatitis symptom scores compared to model controls (SMD = −3.30, 95% CI: −4.37 to −2.23; *p* < 0.001), indicating robust efficacy in ameliorating atopic dermatitis manifestations, as shown in Figure 4.

The heterogeneity variance was estimated at τ^2^ = 2.0083 (95% CI: 0.65–9.80), I^2^ = 84.0% (95% CI: 71.3%–91.1%), indicating significant heterogeneity. The prediction interval [-6.80, 0.20] encompassed the null value of no difference.

##### 3.4.1.2 Subgroup analysis

Subgroup analyses were performed to assess heterogeneity sources and potential treatment effect modifications across key covariates: the AD-induction model, drug formulation, administration method, source, and animal species. There was no statistically significant interaction for drug formulation (*p* for interaction = 0.293), administration method (*p* = 0.321), source (*p* = 0.147), or animal species (*p* = 0.966). These findings possibly indicate that the treatment effect may be consistent across these variables based on the available evidence.

However, several important methodological considerations temper this interpretation. First, the power to detect subgroup differences was likely limited by the relatively small number of studies in each stratum and the overall sample size, especially when comparing groups with fewer than 5–10 studies. Second, substantial residual heterogeneity persisted within most subgroups (I^2^ > 50%), except for DNCB-induced AD models (I^2^ = 35.9%), combination preparations (I^2^ = 43.0%), Zicao (I^2^ = 47.8%), and LE (I^2^ = 40.2%).

In addition, a statistically significant difference was found between AD induction models (*p* for interaction = 0.009), suggesting that treatment efficacy may be influenced by the specific induction model used. However, this finding must be interpreted with caution due to substantial subgroup imbalance in study distribution. Specifically, the DNCB model included seven studies, while the No DNCB model included only two studies. The uneven distribution of studies across the model types may have introduced bias and limited the robustness of this comparison. Moreover, the aforementioned residual heterogeneity within the DNCB subgroup, though lower than that in other strata, still suggests unaccounted variability that may confound the observed model-specific effects.

**FIGURE 5:**
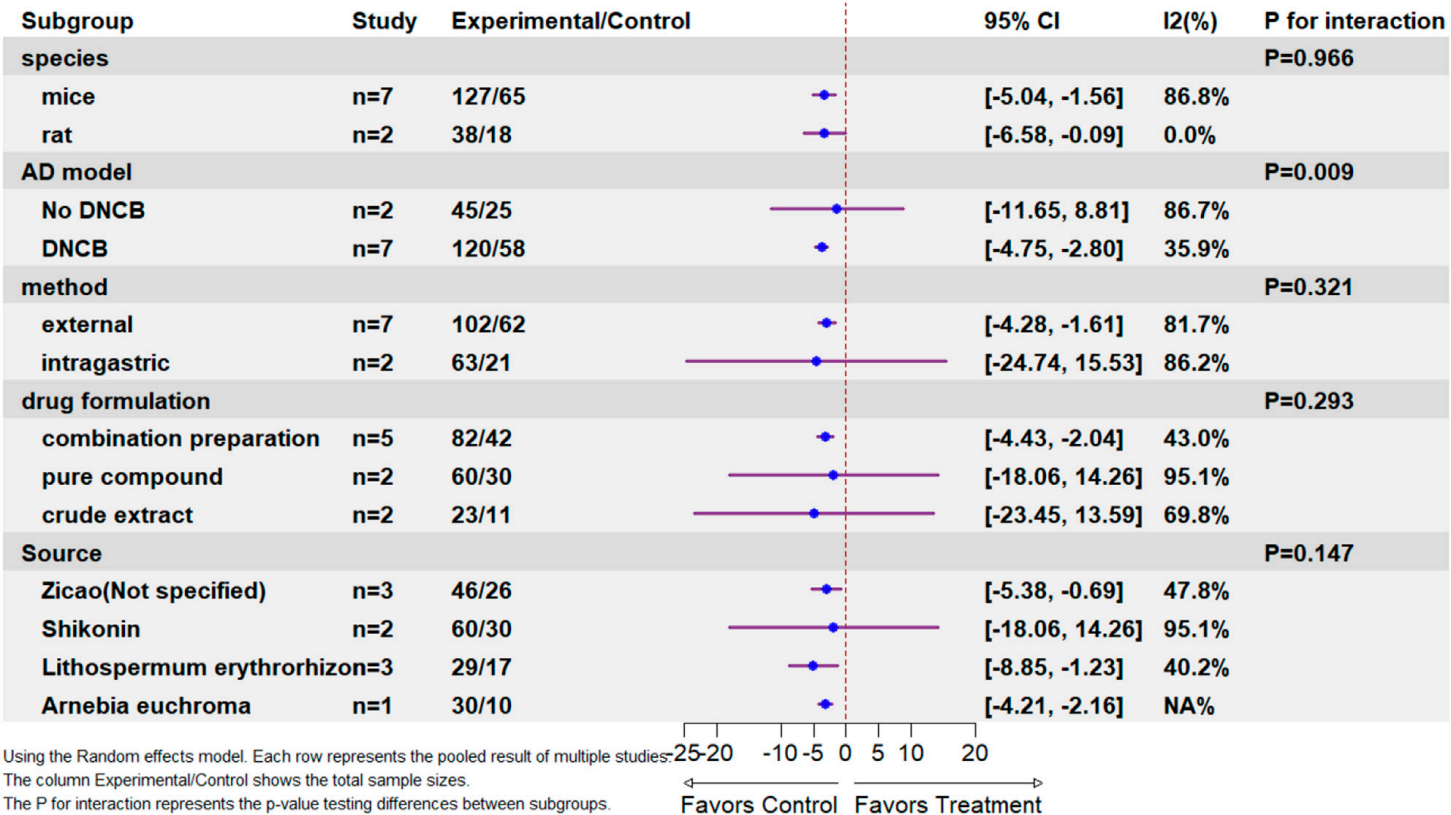
Subgroup analysis.

In conclusion, the collective evidence remains insufficient to attribute therapeutic variations to the species, induction model method formulation, botanical source, or administration differences, and the observed AD model differentiation requires cautious interpretation considering the heterogeneity and subgroup imbalance constraints (Figure 5).

Supplementary: In canonical meta-analytic methods, study weighting is conventionally determined by precision—specifically computed as the inverse of variance estimates. However, in model-based subgroup analyses, weights may be influenced by the model structure and covariates, leading to weight allocations that differ from simple weighted averages and thereby affecting estimates of overall effects. Therefore, the data markers in the figure are presented with a fixed size to represent these conventional weights (Sørensen and Marschner, 2023).

##### 3.4.1.3 Scratching behavior

**FIGURE 6:**
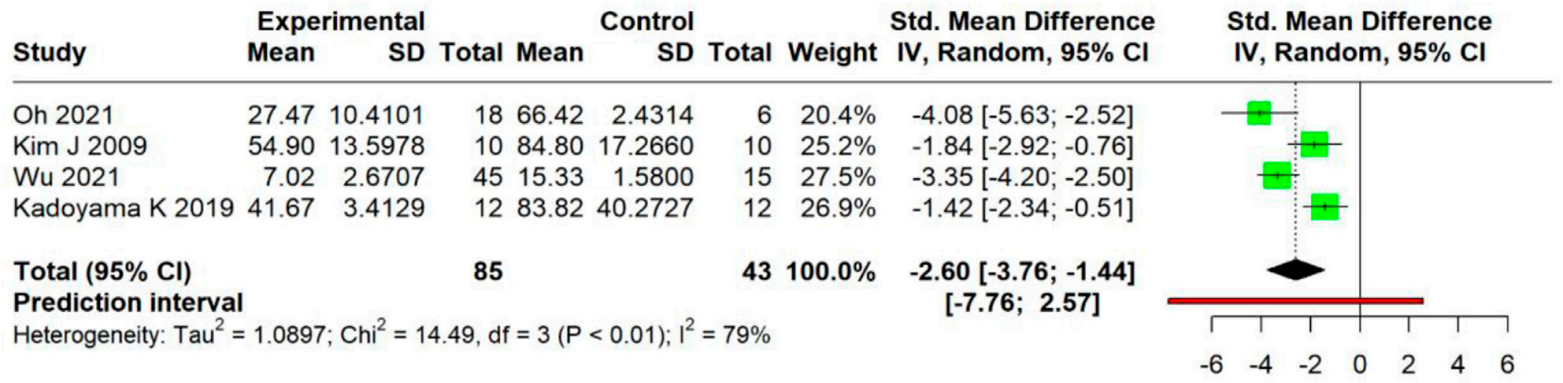
Meta-analysis of scratching behavior.

These studies, using diverse approaches, measured the scratching behavior Oh et al. (2021) and Kim et al. (2009) used a 0–4 scoring system (0 = none; 2 = <1.5 s duration; 4 = >1.5 s duration), summing the observations over 30-minute intervals; Wu et al. (2021) counted discrete scratching bouts (>1 s continuous movement) targeting the dorsal region during 10-minute sessions, while Kadoyama et al. (2019) recorded 1-second scratching episodes on the dorsal/auricular areas over 8 h, reporting the percentage change from baseline. It had a significant effect in reducing scratching behavior (SMD = −2.60, 95% CI: −3.76 to −1.44; *p* < 0.0001), indicating substantial intervention efficacy. However, considerable heterogeneity was observed [τ^2^ = 1.09 (CI: 0.15, 20.10), I^2^ = 79.3% (44.8%; 92.2%)], with the 95% prediction interval spanning from -7.76 to 2.57(Figure 6), and crossing the null value precluded definitive clinical inferences. Due to the small number of research projects, subgroup analysis was not appropriate (Harrer et al., n.d.).

### 3.5 Secondary outcome

The outcomes are organized and presented according to the core pathophysiological axes of AD: beginning with metrics of skin barrier integrity, followed by an analysis of cytokines related to Th1/Th2, and measures of important effector cells and systemic sensitization (mast cells and IgE levels in the serum).

#### 3.5.1 Skin barrier integrity and epidermal remodeling

Epidermal thickness of the dorsal skin was quantified in two studies (Oh et al., 2021; Choi et al., 2017) through microscopic analysis of H&E-stained sections, while the filaggrin (FLG) protein levels in skin tissue were measured by Western blot in two other studies (Oh et al., 2021; Xu et al., 2019).

##### 3.5.1.1 Epidermal thickness of the dorsal skin

**FIGURE 7:**
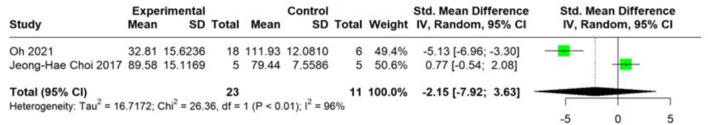
Meta-analysis of the epidermal thickness.

Pooled analysis of two studies (Oh et al., 2021; Choi et al., 2017) indicated no statistically significant effect (SMD = −2.15, 95% CI: −7.92 to 3.63; *p* = 0.47) with extreme heterogeneity (τ^2^ = 16.72; I^2^ = 96.2%, 95% CI: 89.4%–98.6%) (Figure 7).

##### 3.5.1.2 FLG levels

**FIGURE 8:**
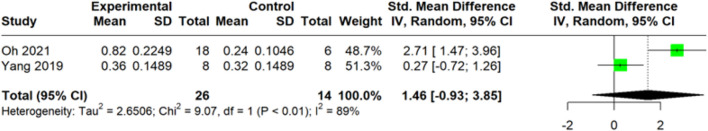
Meta-analysis of the FLG level.

Pooled analysis of two studies (Oh et al., 2021; Xu et al., 2019) suggested no significant increase in FLG level (SMD = 1.46, 95% CI: −0.93 to 3.85; *p* = 0.23) with substantial heterogeneity (τ^2^ = 2.65, 95% CI: 0.84–18.74; I^2^ = 89.0%, 95% CI: 58.5%–97.1%) (Figure 8).

#### 3.5.2 Cytokines related to the Th1/Th2

Across the included studies, the serum protein levels were quantified by the enzyme-linked immunosorbent assay (ELISA) for TNF-α (Oh et al., 2021; Wu et al., 2021), TSLP (Oh et al., 2021; Wu et al., 2021), IL-4 (Oh et al., 2021; Wu et al., 2021; Xiaochao et al., 2019; Xu et al., 2019; Ma et al., 2022), IL-6 (Oh et al., 2021; Ku et al., 2018), and IFN-γ (Wu et al., 2021; Xu et al., 2019; Ma et al., 2022). Meanwhile, skin mRNA expression of TNF-α, IL-4, and IL-13 was measured by reverse transcription quantitative polymerase chain reaction (RT-qPCR) in two studies (Oh et al., 2021; Ku et al., 2018).

##### 3.5.2.1 TNF-α protein levels and TNF-α mRNA expression

**FIGURE 9:**
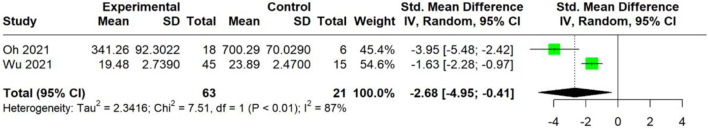
Meta-analysis of TNF-α protein levels (serum).

TNF-α protein levels (serum): The meta-analysis of two studies (Oh et al., 2021; Wu et al., 2021) demonstrated a significant reduction in TNF-α levels following intervention (SMD = −2.68, 95% CI: −4.95 to −0.41; *p* = 0.02), with substantial heterogeneity [τ^2^ = 2.34; I^2^ = 86.7% (95% CI: 47.3%–96.6%)] (Figure 9).

**FIGURE 10:**
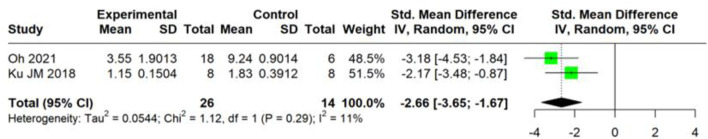
Meta-analysis of TNF-α mRNA expression (skin).

TNF-α mRNA expression (skin): The meta-analysis of two studies (Oh et al., 2021; Ku et al., 2018) revealed a robust decrease in gene expression (SMD = −2.66, 95% CI: −3.65 to −1.67; *p* < 0.0001), supported by minimal heterogeneity (τ^2^ = 0.05; I^2^ = 10.6%) (Figure 10).

##### 3.5.2.2 TSLP protein levels

**FIGURE 11:**
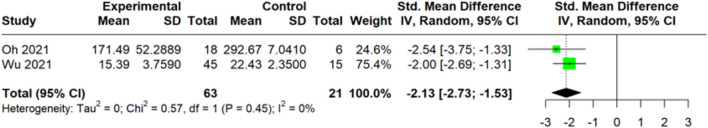
Meta-analysis of TSLP protein levels (serum).

The meta-analysis of two studies (Oh et al., 2021; Wu et al., 2021) demonstrated a significant reduction in the TSLP levels (serum) following intervention (SMD = −2.13, 95% CI: −2.73 to −1.53; *p* < 0.0001), with minimal heterogeneity (τ^2^ = 0; I^2^ = 0%) (Figure 11).

##### 3.5.2.3 IL-4 protein levels and IL-4 mRNA expression

**FIGURE 12:**
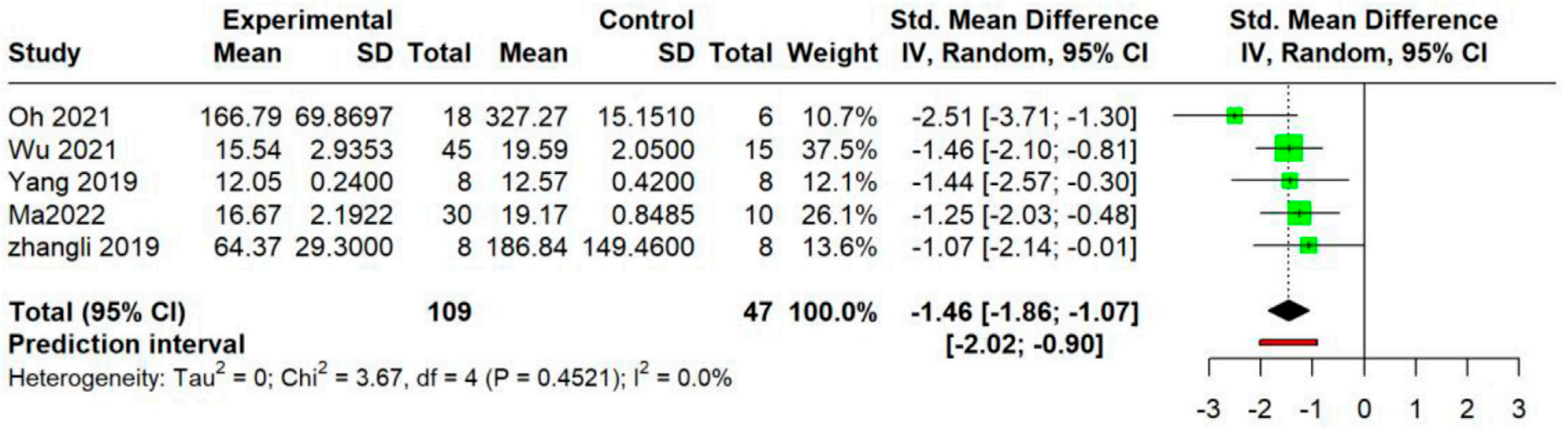
Meta-analysis of IL-4 protein levels (serum).

IL-4 protein levels (serum): The meta-analysis of four studies (Oh et al., 2021; Wu et al., 2021; Liyuan et al., 2019b; Xu et al., 2019; Ma et al., 2022) demonstrated a significant reduction in IL-4 levels following intervention (SMD = −1.46, 95% CI: −1.86 to −1.07; *p* < 0.0001), with low heterogeneity [τ^2^ = 0.0 (CI: 0.00; 1.8782); I^2^ = 0.0% (95% CI: 0.0%–79.2%)]. The prediction interval ranged from −2.02 to −0.90 (Figure 12).

**FIGURE 13:**
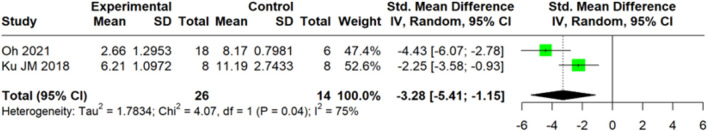
Meta-analysis of IL-4 mRNA expression (skin).

IL-4 mRNA expression (skin): The meta-analysis of two studies (Oh et al., 2021; Ku et al., 2018) demonstrated a significant reduction in IL-4 gene expression following intervention (SMD = −3.28, 95% CI: −5.41 to −1.15; *p* = 0.0025), with substantial heterogeneity [τ^2^ = 1.78; I^2^ = 75.4% (95% CI: 0.0%–94.4%)] (Figure 13).

##### 3.5.2.4 IL-13 mRNA expression

**FIGURE 14:**
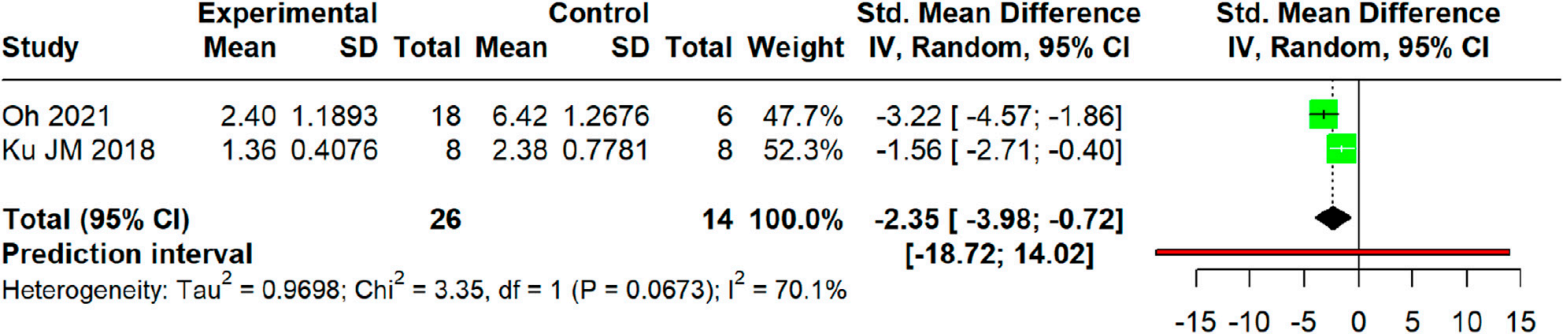
Meta-analysis of IL-13 mRNA expression (skin).

The meta-analysis of two studies (Oh et al., 2021; Ku et al., 2018) demonstrated a significant reduction in IL-13 gene expression (SMD = −2.35, 95% CI: −3.98 to −0.72; *p* = 0.0047) with substantial heterogeneity (τ^2^ = 0.97; I^2^ = 70.1%, 95% CI: 0.0%–93.3%). The prediction interval ranged from −18.72 to 14.02 (Figure 14).

##### 3.5.2.5 IL-6 protein levels

**FIGURE 15:**
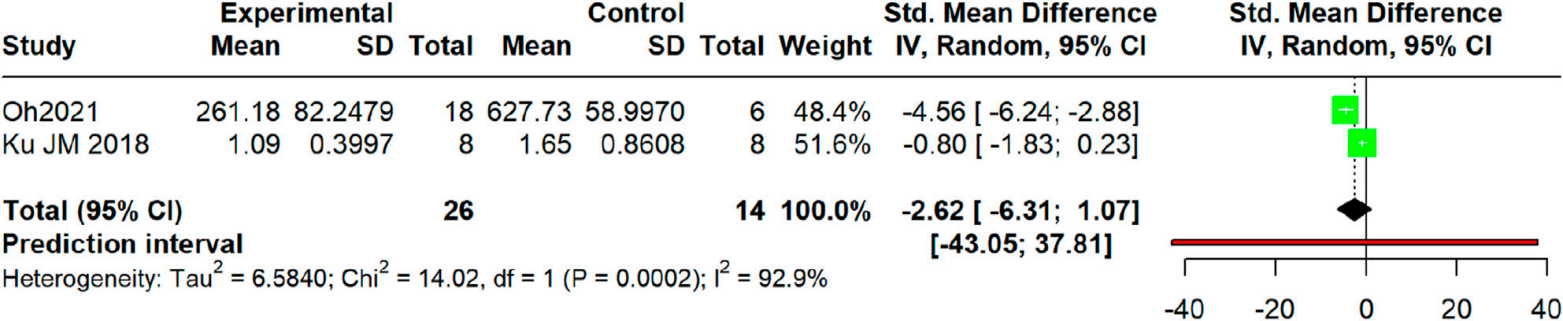
Meta-analysis of IL-6 protein levels (serum).

Pooled analysis of two studies (Oh et al., 2021; Ku et al., 2018) suggested a non-significant reduction in the outcome measure (SMD = −2.62, 95% CI: −6.31 to 1.07; *p* = 0.16) with extreme heterogeneity (τ^2^ = 6.58; I^2^ = 92.9%, 95% CI: 76.2%–97.9%). The prediction interval ranged from −43.05 to 37.81 (Figure 15).

##### 3.5.2.6 IFN-γ protein levels

**FIGURE 16:**
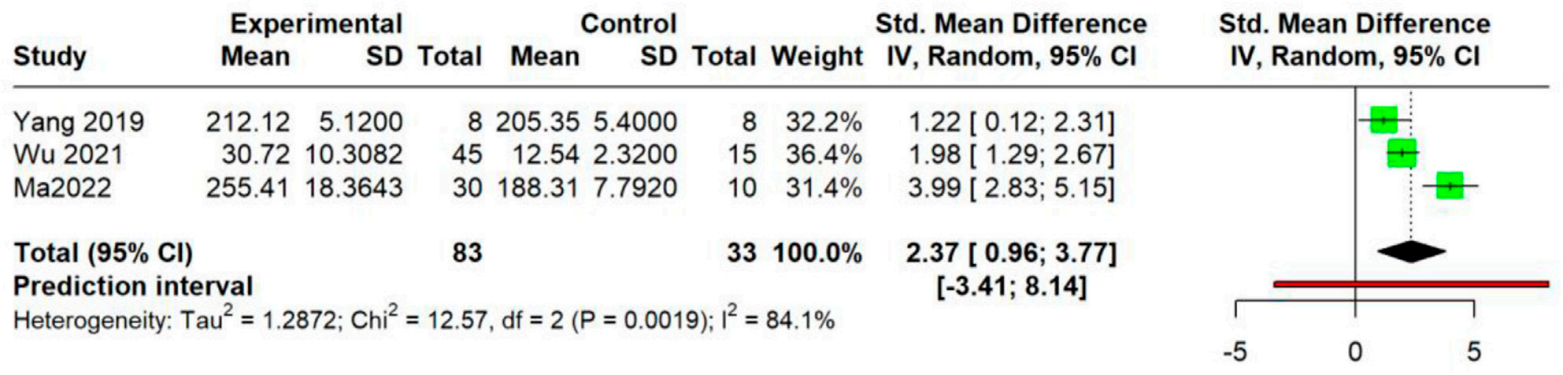
Meta-analysis of IFN-γ protein levels (serum).

Pooled analysis of three studies (Wu et al., 2021; Xu et al., 2019; Ma et al., 2022) indicated a statistically significant effect (SMD = 2.37, 95% CI: 0.96 to 3.77; *p* = 0.001). Substantial heterogeneity was observed (τ^2^ = 1.2872, 95% CI: 0.2261–70.1486; I^2^ = 84.1%, 95% CI: 52.3%–94.7%). The prediction interval ranged from −3.41 to 8.14 (Figure 16).

#### 3.5.3 Mast cells and IgE levels

Mast cell counts in the skin tissue, quantified from toluidine blue-stained sections in two studies (Oh et al., 2021; Ku et al., 2018), demonstrated a significant reduction. In addition, IgE levels in the serum were measured using ELISA in four studies (Oh et al., 2021; Ku et al., 2018; Lee et al., 2009; Kim et al., 2009).

##### 3.5.3.1 Mast cells count

**FIGURE 17:**
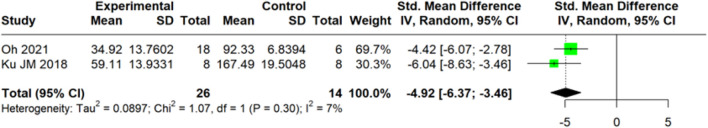
Meta-analysis of mast cell level.

Pooled analysis of two studies (Oh et al., 2021; Ku et al., 2018) that quantitatively assessed mast cell counts in skin tissue demonstrated a significant reduction (SMD = −4.92, 95% CI: −6.37 to −3.46; *p* < 0.0001) with minimal heterogeneity (τ^2^ = 0.09; I^2^ = 6.8%) (Figure 17).

##### 3.5.3.2 IgE protein levels

**FIGURE 18:**
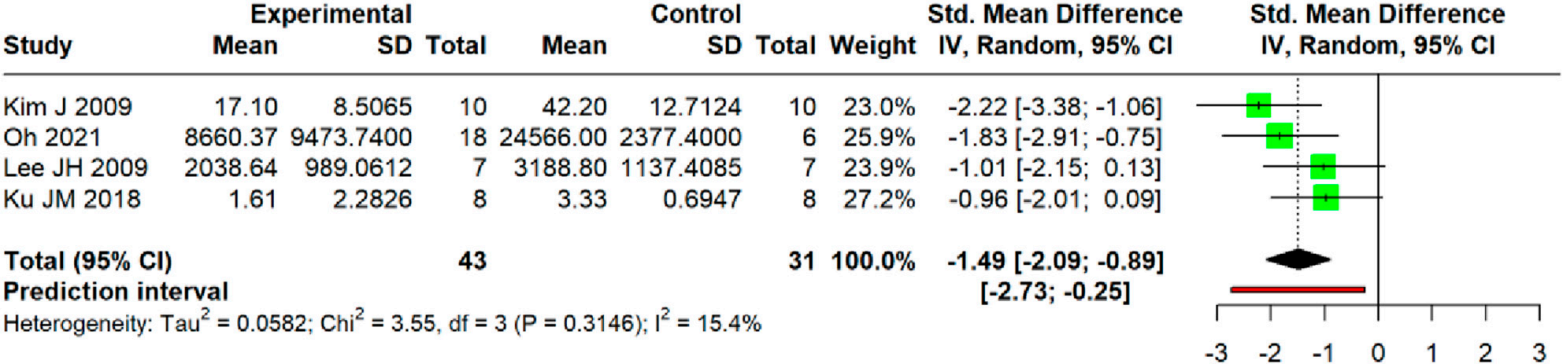
Meta-analysis of IgE level (serum/skin).

Pooled analysis of four studies (Oh et al., 2021; Ku et al., 2018; Lee et al., 2009; Kim et al., 2009) demonstrated a significant reduction in mast cell biomarkers (SMD = −1.49, 95% CI: −2.09 to −0.89; *p* < 0.0001). Heterogeneity variance was estimated at τ^2^ = 0.0582 (95% CI: 0.00–4.9805), with I^2^ = 15.4% (95% CI: 0.0%–87.1%). The prediction interval ranged from −2.73 to −0.25 (Figure 18).

### 3.6 Influence analysis and publication bias

For SCORAD and EASI, the influence diagnostics via R-based outlier assessment and leave-one-out sensitivity analysis identified Kadoyama et al. (2019) as the primary contributor to substantial heterogeneity. Exclusion of these outliers yielded a marked reduction in heterogeneity indices: I^2^ decreased from 84.0% to 55.5%, while τ^2^ = 0.5931 (95% CI: 0.0000–5.6277) encompassed the null value of 0, and Cochran’s Q heterogeneity test remained statistically significant (*p* = 0.0278).

In addition, elimination of studies with disproportionate effect weights (SMD = −3.5431, CI: 4.3086, -2.7776; *p* < 0.0001) did not significantly alter the primary outcome trajectory, thus further validating the result stability (Supplementary Material).

**FIGURE 19:**
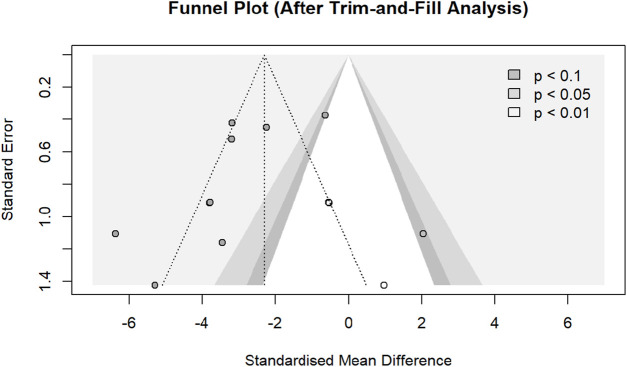
Enhanced funnel plot analysis. Note: Imputed studies are shown as unfilled circles.

Enhanced funnel plot analysis revealed asymmetry, with Egger’s test indicating potential subtle publication bias (intercept = −4.057, 95% CI: −6.95 to −1.16; *p* = 0.0286). Trim-and-fill imputation added four studies: one significant study (Oh 2021-like) at *p* < 0.1 and three non-significant studies in the funnel’s midzone, yielding an adjusted estimate of SMD = −2.2983 (95% CI: −3.31 to −1.2847; *p* < 0.0001; I^2^ = 84.8%) (Figure 19).

## 4 Discussion

### 4.1 Summary of the results

This systematic review and meta-analysis includes data from 12 studies involving 316 animals, evaluating the therapeutic profile of Zicao and its primary bioactive naphthoquinones (e.g., shikonin) in experimental models of AD.

The primary outcomes demonstrated significant treatment effects on the dermatitis severity scores (SMD = −3.30, I^2^ = 84.0%) and scratching behavior (SMD = −2.60, I^2^ = 79.3%). However, the prediction intervals for both crossed the null value (dermatitis: −6.80 to 0.20; scratching: −7.76 to 2.57), indicating that while the pooled estimates indicate substantial average efficacy, the effects in the subsequent studies may vary from significantly beneficial to null or oppositely directed under varying experimental conditions. Subgroup analyses of dermatitis severity scores indicated the AD induction model as a statistically significant effect modifier (*p* = 0.009), while DNCB-induced models demonstrated reduced heterogeneity (I^2^ = 35.9%). However, the uneven distribution of studies among subgroups and limited statistical power precluded definitive conclusions regarding the influence of the formulation, route of administration, botanical source, or host species. Sensitivity analysis excluding one influential outlier reduced heterogeneity to I^2^ = 55.5% while maintaining significant effects (SMD = −3.5431, CI: 4.3086,-2.7776; *p* < 0.0001). Trim-and-fill adjustment for detected publication bias (Egger’s test, *p* = 0.0286) yielded a more conservative, though still statistically significant, estimate (adjusted SMD = −2.30, *p* < 0.0001), supporting the general robustness of the primary findings.

For IL-4, a highly consistent inhibitory effect was observed at both the protein level in the serum (SMD = −1.46, I^2^ = 0%) and the mRNA level in skin tissue (SMD = −3.28, I^2^ = 75.4%). The former analysis demonstrated lower heterogeneity, with a prediction interval (−2.02 to −0.90) that entirely excluded 0 and remained below the null value, suggesting that future individual studies are likely to yield effects in the same direction, whereas the latter analysis revealed a larger effect size alongside substantial heterogeneity. Similarly, a potent and homogeneous reduction in TSLP was confirmed in the serum (SMD = −2.13, I^2^ = 0%). In stark contrast, the effect on IL-13 mRNA, while significant on average (SMD = −2.35), was markedly heterogeneous (I^2^ = 70.1%) with a prediction interval (−18.72 to 14.02); this wide-spanning interval, which crosses the null value, indicates the potential for future studies to demonstrate null or even opposing effects. TNF-α was reduced at the mRNA level in the skin (SMD = −2.66, I^2^ = 10.6%) and the protein level in the serum (SMD = −2.68, I^2^ = 86.7%), whereas IL-6 exhibited non-significant effects (SMD = −2.62, *p* = 0.16) with extreme heterogeneity (I^2^ = 92.9%; prediction interval: −43.05 to 37.81), rendering the pooled estimate uninterpretable. Conversely, an increase in IFN-γ protein was observed (SMD = 2.37, *p* = 0.001; I^2^ = 84.1%) with a prediction interval (−3.41–8.14); the pronounced heterogeneity combined with a null-crossing prediction interval suggests that this finding may lack robustness, and future individual studies may plausibly demonstrate null or contradictory results.

### 4.2 Evidence for the clinical efficacy

Beyond the preclinical evidence presented in our meta-analysis, some clinical studies indicated the therapeutic potential of Zicao (the root of LE Siebold and Zucc., *Arnebia euchroma* (Royle) Johnst., or *Arnebia guttata* Bunge) in AD. A clinical trial found that an ointment containing shikonin significantly improved dermatitis and reduced the EASI score in AD patients (Yen and Hsieh, 2016). In addition, the same research team used human dendritic cells derived from AD patients and showed that shikonin potently suppressed the expression of multiple allergen-induced pro-inflammatory cytokines and chemokines, with its inhibitory effect on IL-9, MIP-1β, and RANTES exceeding that of dexamethasone (Chung-Yang et al., 2020). In addition, a randomized controlled trial by Cho et al. (2008) involving 28 AD subjects showed that oral administration of LE extract (1.5 g daily for 10 weeks) significantly increased stratum corneum hydration and ceramide levels compared to that with placebo, with improvements positively correlating with baseline disease severity. Another clinical trial also indicated that a compound Zicao oil preparation had comparable efficacy to pimecrolimus for symptomatic relief, alongside a more favorable safety profile (Liyuan et al., 2019a).

It is worth noting that the barrier repair effects observed in the human trial by Cho et al. (2008) appear to contrast with the insignificant findings for epidermal thickness and filaggrin expression in our meta-analysis. This discrepancy may be attributed to several factors, including potentially limited sample sizes and animal model differences in skin physiology and disease manifestation.

### 4.3 Heterogeneity in AD animal models and its potential impact on the treatment response

The variety of AD modeling methods, each focusing on different pathophysiological mechanisms, represents a potential confounding factor affecting therapeutic efficacy outcomes. The substantial heterogeneity observed across most indicators in this meta-analysis, along with subgroup analyses of the primary outcome measure (dermatitis score), suggests that AD animal modeling is a statistically significant effect modifier. It requires an extensive examination of modeling methodologies.

There are three main types of AD animal models: The first category consists of hapten-induced models, which use repeated applications of haptens [oxazolone, DNCB (2,4-dinitrochlorobenzene)] to induce a transition from delayed-type hypersensitivity to Th2-dominated chronic inflammation, characterized by elevated levels of Th2-associated cytokines, including IL-4, IL-5, and IL-13. The widely used DNCB-induced AD model causes disease by directly stimulating keratinocytes and sensory neurons and causing keratinocytes to release cytokines and chemokines. The molecular mechanisms behind this include the mitogen-activated protein kinase (MAPK)/nuclear factor-κB (NF-κB) and erythroid 2-related factor 2 (Nrf2)/heme oxygenase-1 (HO-1) signaling pathways, along with the involvement of several immune cell types, such as Th1, Th2, mast cells, and eosinophils.

The oxazolone (OX)-induced model primarily operates through the TRPV1, MAPK-AP1, and ROS pathways to promote inflammation. While DNCB modeling emphasizes MAPK/NF-κB signaling, OX modeling predominantly activates the MAPK-AP1 pathway (Jin et al., 2009).

The second category includes allergen-induced models, such as ovalbumin (OVA) and house dust mite (HDM) models, wherein the OVA-induced AD model demonstrates relative independence from the mouse strain, age, and sex, exhibiting a notable immunological shift from Th2 to a mixed Th1 + Th2 response and involving IgE-independent mast cell activation pathways. The HDM model induces epidermal and dermal thickening, increased infiltration of mast cells and eosinophils, and downregulation of skin barrier proteins (filaggrin and loricrin), with inflammatory responses being significantly amplified when combined with barrier disruption protocols, and NC/Nga mice are frequently used for this modeling approach (Sanjel and Shim, 2022).

The third category consists of spontaneous models, represented by the NC/Nga mouse strain, which develops pruritic skin lesions spontaneously at approximately 8 weeks of age under conventional housing conditions, exhibiting impaired water-retention properties and barrier dysfunction, reduced ceramide content, intrinsic susceptibility to AD-like dermatitis, and high expression of Th2-specific chemokines, including thymus and activation-regulated chemokine (TARC) and macrophage-derived chemokine (MDC), along with their receptor CCR4 in lesional skin (Zheng et al., 2024).

The use of DNCB-induced models utilizing non-BALB/c mouse strains in the current analysis may have contributed to the observed heterogeneity. A critical interspecies discrepancy requires attention: while human AD patients demonstrate FLG gene downregulation in lesional skin, FLG expression is paradoxically upregulated in NC/Nga and OVA models (Ewald et al., 2017), which may explain the discordance between the clinical study (Cho et al., 2008) showing significant FLG upregulation following Zicao treatment and the non-significant results observed in this meta-analysis. Since Zicao may primarily exert its therapeutic effects via Th2 modulation and its associated mechanisms, increased efficacy is expected in Th2-dominant models.

### 4.4 Possible mechanisms

#### 4.4.1 Control of the Th2 inflammatory effect

The Th2 immune response is fundamental within the pathogenic processes of AD. IL-4 and IL-13 are the main cytokines that drive Th2 immunity. They promote Th2 cell differentiation and IgE class switching in B cells through signaling pathways such as JAK/STAT. Furthermore, studies indicate that IL-4 and IL-13 induce characteristic epidermal pathology in AD, including downregulation of E-cadherin expression (Ohtani et al., 2009), accumulation of hyaluronic acid, and intercellular space widening (spongiosis).

TNF-α also significantly contributes to inflammation perpetuation and chronicity. Vakirlis et al. (2011) identified a correlation between elevated TNF-α levels and increased disease severity. Moreover, a study using the Leiden epidermal model (LEM), which recapitulates AD features, demonstrated that TNF-α acts synergistically with Th2 cytokines (e.g., IL-4 and IL-13) to induce spongiosis in the LEM, enhance TSLP secretion by keratinocytes, alter the expression of early and terminal differentiation proteins, and reduce ceramide levels (Danso et al., 2014).

TSLP is widely regarded as a key upstream initiator (alarmin) in the atopic immune response characteristic of AD. TSLP can be released through the ORAI1/NFAT calcium signaling pathway when epidermal keratinocytes are damaged. In addition to its well-known function of stimulating Th2 cell differentiation by causing dendritic cells (DCs) to express OX40L, TSLP also directly stimulates Th2 polarization in CD4^+^ T cells. Notably, TSLP accomplishes this by triggering the transcription of the *IL-4* gene, which is independent of STAT6 signaling and results in the production of IL-4 (Kitajima et al., 2011; Omori and Ziegler, 2007) However, STAT6 continues to be necessary for the differentiation of Th2 cells. Additionally, TSLP and IL-33 work together to activate type-2 innate lymphoid cells (ILC2s), which increases IL-5 and IL-13 production and intensifies inflammatory responses (Ryffel and Alves-Filho, 2019).

Chinese traditional herb Zicao and its bioactive constituents have been demonstrated to modulate Th2 inflammation-associated mechanisms. A study reported that reflux ethanolic extracts of LE significantly reduced the levels of IL-4 and IL-13 in nasal lavage fluid obtained from murine models of allergic rhinitis. Furthermore, this study also identified N,N′-dicoumaroyl spermidine, the principal bioactive compound present in the extract, as an inhibitor of IL-4 and IL-13 expression in bone marrow-derived mast cells (Le et al., 2022). Another study investigating the effects of an aqueous extract of LE on inflammatory responses induced by *Dermatophagoides pteronyssinus* group-2 allergen (Der p2) in human bronchial epithelial cells (BEAS-2B) demonstrated that the extract inhibited the production of TSLP (Yen et al., 2017). Additionally, a different study demonstrated that LE extract reduced the expression of tumor necrosis factor TNF-α, IL-6, and IL-8 in lipopolysaccharide (LPS)-stimulated macrophages through the inhibition of nuclear NF-κB/AP-1 and IRF signaling pathways (Kang et al., 2023).

A limitation in the current literature is that the anti-inflammatory properties of *Arnebia euchroma* have not been fully elucidated at a mechanistic level (Kumar et al., 2021).

More studies have focused on shikonin, the principal bioactive constituent of Zicao. Shikonin has been shown to alleviate excessive Th2 cell activation in rat models of allergic rhinitis by reducing the expression of co-stimulatory molecules CD80 and CD86 on dendritic cells (Liu and You, 2016) while concurrently reducing the circulating levels of IL-4 in peripheral blood (Li and You, 2018). Another research study examined the effects of shikonin on type-2 cytokine production in Jurkat T cells. This investigation revealed that shikonin suppresses PMA/cAMP-induced IL-4 mRNA and protein expression through the downregulation of the transcription factors GATA binding protein 3 (GATA-3) and c-musculoaponeurotic fibrosarcoma oncogene homolog (c-Maf) (Lee et al., 2011).

Furthermore, Th2 cell differentiation is regulated by multiple signal transducer and activator of transcription (STAT) proteins. This process is dependent on STAT6, and in the presence of IL-4, STAT3 and STAT6 can synergistically promote Th2 differentiation (Stritesky et al., 2011). Clinical investigations have demonstrated that pediatric patients with AD exhibited elevated serum STAT3 levels, which demonstrated a significant positive correlation with the SCORAD index (Lyu et al., 2014), thereby confirming the pathogenic relevance of STAT3 in human disease. In addition, a separate study utilizing OVA/HDM-sensitized murine asthma models demonstrated that shikonin suppresses STAT3 expression levels in the airway epithelium (Zhang et al., 2024). Additionally, research examining TNF-α regulation has revealed that shikonin interferes with the basal transcriptional machinery to prevent the transcriptional activation of the human TNF-α promoter (Staniforth et al., 2004).

The meta-analysis also observed an increase in IFN-γ levels following Zicao intervention, which might suggest a potential modulatory effect on IFN-γ expression in the context of AD. IFN-γ, a canonical Th1 cytokine, functions through transcription factors including T-bet, STAT1, and STAT4 to promote Th1 differentiation while simultaneously suppressing Th2 responses. However, it is important to note that IFN-γ has also been implicated in epidermal barrier disruption by modifying the fatty acid composition of ceramides (Brar and Leung, 2016). A study reported that shikonin may enhance IFN-γ production, possibly contributing to Th1/Th2 recalibration (Zhang et al., 2024).

#### 4.4.2 Regulation of mast cell activation and pruritus signaling

IgE, mast cells (MCs), and TSLP (AD) contributed to both the inflammatory response and pruritic manifestations of AD. IgE and MCs constitute critical mediators of inflammatory immune responses in AD. When MCs are activated, they release a wide range of mediators that control the recruitment, migration, and functional activity of inflammatory cells in cutaneous tissues. Th2 cell activation and differentiation are aided by IL-4 and IL-13 released by activated MCs. The histamine released from MCs induces the expression of adhesion molecules, pro-inflammatory cytokines, and chemokines by keratinocytes (Liu et al., 2011). Furthermore, FcεRI-mediated MC activation triggers the production of prostaglandin D2 (PGD_2_), which promotes the migration of group-2 innate lymphoid cells (ILC2s) to cutaneous sites and stimulates type-2 cytokine synthesis through signaling via its cognate receptors (DP1/DP2) (Mortaz et al., 2018).

The binding of IgE to the high-affinity IgE receptor (FcεRI) on the surface of mast cells (MCs) sensitizes these cells. Subsequent cross-linking of these IgE molecules by a specific allergen triggers MC degranulation, leading to the release of histamine and other mediators that induce pruritus (itch).

Additionally, through direct neuronal mechanisms, TSLP contributes to pruritus associated with AD. According to experimental data, TSLP causes acute itching behaviors by directly activating a subset of transient receptor potential ankyrin 1-positive (TRPA1^+^) sensory neurons (Wilson et al., 2013).

In addition to histamine-mediated pruritus, MCs can elicit antihistamine-resistant itching through alternative pathways. Activation of MRGPRX2 (human)/MRGPRB2 (murine) receptors on MCs leads to the release of proteases, including tryptases, which act upon proteinase-activated receptor 2 (PAR-2) expressed on the sensory neurons. Moreover, investigations utilizing murine AD models indicate that protease release mediated by MRGPRX2/MRGPRB2 activation also influences type-2 cytokine production (Jia et al., 2024).

Current studies demonstrate that shikonin suppresses mucosal MC activation by inhibiting calcineurin activity and subsequently reducing the expression of the Nr4a family genes (Wang et al., 2014).

Concurrently, an extract of LE was shown to inhibit histamine release from rat peritoneal MCs in a dose-dependent manner (Kim et al., 2007). Further evidence indicates that shikonin specifically interacts with and inhibits C48/80-induced Mrgprx2 expression in human embryonic kidney (HEK) cells (Wang et al., 2020).

The results of a clinical study on patients with STAT3 mutations may be pertinent given the known inhibitory effect of shikonin on STAT3 expression in the airway epithelium of murine asthma models (discussed previously) and the dual function of STAT3 in MC degranulation. According to this study, STAT3 is crucial for proximal signaling events that are necessary for IgE-dependent MC degranulation, but its function in IgE-independent degranulation appears to be less significant (Siegel et al., 2013).

#### 4.4.3 Effects on Cutaneous Integrity and Barrier Function

This meta-analysis found no statistically significant difference in epidermal thickness following intervention with Zicao or its active components. This observation suggests a potentially limited effect on epidermal hyperplasia (acanthosis) or hyperkeratosis in animal models of AD. Notably, there are contrasting findings regarding psoriasis, a distinct Th1/Th17-skewed inflammatory skin disorder where evidence indicates that LE components can suppress keratinocyte hyperproliferation and promote apoptosis (Wang et al., 2022).

Furthermore, the meta-analysis demonstrated no significant change in FLG protein expression post-intervention.

### 4.5 Limitations

This systematic review has inherent limitations that necessitate cautious interpretation of the findings. First, critical methodological deficiencies were identified via SYRCLE assessment—inadequate randomization reporting (only 3/12 studies described sequence generation), universal lack of allocation concealment and blinding, and unaccounted animal exclusions post-randomization in one study—collectively inflating efficacy estimates. Second, substantial unexplained heterogeneity persisted in primary outcomes, which may be attributable to variable disease induction methods, inconsistent outcome measurement methods, and divergent interventions (oral vs. topical administration; whole-herb vs. isolated compounds). Third, pharmacological standardization was severely compromised by the universal absence of phytochemical quantification (e.g., shikonin/acetylshikonin concentrations) and inadequate botanical documentation (voucher specimens and extraction protocols), precluding dose–response interpretation. Finally, critical translational gaps exist: none of the studies reported pharmacokinetics, toxicology profiles, adverse events, or long-term efficacy data. Furthermore, to maintain methodological rigor, our quantitative synthesis was restricted to outcome measures that were consistently reported in at least two independent studies. Consequently, several potentially important parameters, such as specific cell-signaling pathway components and microbiome composition, could not be evaluated due to insufficient reporting.

## 5 Conclusion

This meta-analysis synthesizes the existing preclinical evidence on the Chinese traditional herb Zicao and its bioactive components for AD. The results indicate possible advantages in alleviating dermatitis severity and pruritus, likely driven by the modulation of Th2 immunity and mast cell activation. However, the pooled effect estimates from this meta-analysis should be interpreted as preliminary findings of potential efficacy rather than definitive evidence, given the pervasive inadequacy of methodological reporting across the included studies. To build a credible evidence base for any future clinical consideration, studies must first prioritize rigorous and standardized preclinical research, including: 1) conducting chemical profiling of extracts using guidelines such as ConPhyMP; 2) implementing and reporting randomization, blinding, and sample-size calculations in accordance with the ARRIVE guidelines; 3) performing systematic dose–response studies; and 4) incorporating comprehensive toxicological and safety assessments.

## Data Availability

The original contributions presented in the study are included in the article/Supplementary Material; further inquiries can be directed to the corresponding author.
