# Supplementary material for: A meta-analysis of the traditional herb Zicao and its active components for atopic dermatitis
Source: Front Pharmacol. 2026 Jan 6;16:1648894. doi: 10.3389/fphar.2025.1648894 (PMC12815445; doi:10.3389/fphar.2025.1648894)
Supplement: Supplementary file 1 [file Supplementaryfile1.docx]

Supplementary Material

Material S1. Search strategy in PubMed

**#1**

(((((((lithospermum[MeSH Terms]) OR (Lithospermum[Title/Abstract])) OR (Lithosperm*[Title/Abstract])) OR (Puccoon*[Title/Abstract])) OR (Gromwell*[Title/Abstract])) OR (Radix Arnebiae (Zi Cao[Title/Abstract]))) OR (Radix Arnebiae[Title/Abstract])) OR (Zi Cao[Title/Abstract])

1,055 resualt

**#2**

((((((((((((("shikonin"[Supplementary Concept]) OR ("Acetylshikonin"[Supplementary Concept])) OR ("beta,beta-dimethylacrylshikonin"[Supplementary Concept])) OR

("Isovalerylshikonin"[Supplementary Concept])) OR ("beta-hydroxyisovalerylshikonin"[Supplementary Concept])) OR ("Deoxyshikonin"[Supplementary Concept])) OR ("Arnebinone"[Supplementary Concept])) OR ("alkannin"[Supplementary Concept])) OR ("Propionylshikonin"[Supplementary Concept])) OR ("(2-methyl-n-butyl)shikonin"[Supplementary Concept])) OR ("5,8-dihydroxy-2-(1'-cinnamoyloxy-4'-methylpent-3'-enyl)-1,4-naphthoquinone"[Supplementary Concept])) OR ("1-(5,8-dihydro-1,4-dihydroxy-5,8-dioxo-2-naphthyl)-4-methylpent-3-en-1-yl cinnamate"[Supplementary Concept])) OR ("beta,beta-dimethylacryloyl alkannin"[Supplementary Concept])) OR ("beta-acetoxyisovalerylshikonin"[Supplementary Concept])

898 resualt

**#3**

((((((((("Boraginaceae"[MeSH Terms]) OR (Boraginaceae[Title/Abstract])) OR (Cynoglossum[Title/Abstract])) OR (Cynoglossums[Title/Abstract])) OR (Arnebia*[Title/Abstract])) OR (Alkanna*[Title/Abstract])) OR (Onosma*[Title/Abstract])) OR (Tournefortia*[Title/Abstract])) OR (Ehretia*[Title/Abstract])) OR (Anchusa*[Title/Abstract])

2055 resualt

**#4**

(((((((((((((((((((((("1,4-naphthalenedione,5,8-dihydroxy-2-((1R)-1-hydroxy-4-methyl-3-penten-1-yl)-"[Title/Abstract]) OR ("isoarnebin 4"[Title/Abstract])) OR ("1,4-naphthalenedione,5,8-dihydroxy-2-(1-hydroxy-4-methyl-3-pentenyl)-, (R)-"[Title/Abstract])) OR ("1,4-naphthalenedione,5,8-dihydroxy-2-((1r)-1-hydroxy-4-methyl-3-pentenyl)-"[Title/Abstract])) OR ("2-((1R)-1-hydroxy-4-methyl-3-pentenyl)-5,8-dihydroxy-1,4-naphthoquinone"[Title/Abstract])) OR ("shikonin, (+)-isomer"[Title/Abstract])) OR ("(+)-shikonin"[Title/Abstract])) OR ("(R)-(+)-shikonin"[Title/Abstract])) OR ("5,8-dihydroxy-2-((1R)-1-hydroxy-4-methyl-3-penten-1-yl)-1,4-naphthalenedione"[Title/Abstract])) OR ("1,4-naphthoquinone,5,8-dihydroxy-2-(1-hydroxy-4-methyl-3-pentenyl)-, (+)-"[Title/Abstract])) OR ("(+-)-shikonin"[Title/Abstract])) OR ("5,8-dihydroxy-2-(1-hydroxy-4-methyl-3-pentenyl)-1,4-naphthalenedione"[Title/Abstract])) OR ("acetylshikonin, (+-)-isomer"[Title/Abstract])) OR ("acetylshikonin, (S)-isomer"[Title/Abstract])) OR ("acetylshikonin, (R)-isomer"[Title/Abstract])) OR ("(1R)-1-(5,8-Dihydroxy-1,4-dioxo-1,4-dihydro-2-naphthalenyl)-4-methyl-3-penten-1-yl acetate"[Title/Abstract])) OR ("arnebin I"[Title/Abstract])) OR ("DMASK"[Title/Abstract])) OR ("beta, beta-dimethylacrylshikonin, (-)-isomer"[Title/Abstract])) OR ("beta, beta-dimethylacrylshikonin, (+)-isomer"[Title/Abstract])) OR ("beta-HIVS"[Title/Abstract])) OR (shikonin analog 93-637[Title/Abstract])) OR (SA 93-637[Title/Abstract])

1,259 resualt

**#5**

#2 OR #4 1,382 resualt

**#6**

#1 OR #3 2,799 resualt

**#7**

#5 OR #6 3,626 resualt

**#8**

Shikonofuran*[Title/Abstract] 15 resualt

**#9**

#7 OR #8 3,628 resualt

**#10**

((((((((((((((((Dermatitis, Atopic[Title/Abstract]) OR (Atopic Dermatitides[Title/Abstract])) OR (Atopic Dermatitis[Title/Abstract])) OR (Dermatitides, Atopic[Title/Abstract])) OR (Neurodermatitis, Atopic[Title/Abstract])) OR (Atopic Neurodermatitides[Title/Abstract])) OR (Atopic Neurodermatitis[Title/Abstract])) OR (Neurodermatitides, Atopic[Title/Abstract])) OR (Neurodermatitis, Disseminated[Title/Abstract])) OR (Disseminated Neurodermatitides[Title/Abstract])) OR (Disseminated Neurodermatitis[Title/Abstract])) OR (Neurodermatitides, Disseminated[Title/Abstract])) OR (Eczema, Atopic[Title/Abstract])) OR (Atopic Eczema[Title/Abstract])) OR (Eczema, Infantile[Title/Abstract])) OR (Infantile Eczema[Title/Abstract])) OR (Dermatitis, Atopic[MeSH Terms])

41,709 resualt

**#11**

#9 AND #10 18 resualt

Material S2. Search strategy in Web of Science

# Searches:

1: TS=('Dermatitis, Atopic' OR Atopic Dermatitides OR Atopic Dermatitis OR 'Dermatitides, Atopic' OR 'Neurodermatitis, Atopic' OR Atopic Neurodermatitides OR Atopic Neurodermatitis OR 'Neurodermatitides, Atopic' OR 'Neurodermatitis, Disseminated' OR Disseminated Neurodermatitides OR Disseminated Neurodermatitis OR 'Neurodermatitides, Disseminated' OR 'Eczema, Atopic' OR Atopic Eczema OR 'Eczema, Infantile' OR Infantile Eczema) and Preprint Citation Index (Exclude – Database) Date Run: Tue Jun 17 2025 21:00:14 GMT+0800 (中国标准时间) Results: 66139

2: TS=('Gromwell*' OR Lithosperm* OR 'Puccoons' OR 'Puccoon') and Preprint Citation Index (Exclude – Database) Date Run: Tue Jun 17 2025 21:01:29 GMT+0800 (中国标准时间) Results: 1989

3: TS=('Tournefortia*' OR 'Cynoglossum*' OR 'Anchusa*' OR 'Alkanna*' OR 'Onosma*' OR 'Ehretia*' OR 'Arnebia*' OR Zicao OR 'Boraginaceae') and Preprint Citation Index (Exclude – Database) Date Run: Tue Jun 17 2025 21:01:54 GMT+0800 (中国标准时间) Results: 7458

4: TS=(shikonin* OR 'Acetylshikonin' OR 'beta,beta-dimethylacrylshikonin' OR 'Isovalerylshikonin' OR 'beta-hydroxyisovalerylshikonin' OR 'Deoxyshikonin' OR 'Arnebinone' OR alkannin* OR 'Propionylshikonin' OR 'beta,beta-dimethylacryloyl alkannin' OR 'beta-acetoxyisovalerylshikonin' 'isoarnebin 4' OR 'arnebin I' OR 'DMASK') and Preprint Citation Index (Exclude – Database) Date Run: Tue Jun 17 2025 21:02:29 GMT+0800 (中国标准时间) Results: 2147

5: #2 OR #3 OR #4 and Preprint Citation Index (Exclude – Database) Date Run: Tue Jun 17 2025 21:03:08 GMT+0800 (中国标准时间) Results: 9616

6: #5 AND #1 and Preprint Citation Index (Exclude – Database) Date Run: Tue Jun 17 2025 21:04:01 GMT+0800 (中国标准时间) Results: 43

Material S3. Search strategy in Embase

No. Query Results Results Date

#8. #1 AND #7 38 17 Jun 2025

#7. #2 OR #3 OR #4 OR #5 OR #6 5,826 17 Jun 2025

#6. (((((((((((((((('1,4 naphthalenedione,5,8 2 17 Jun 2025

dihydroxy 2':ti,ab,kw AND 1r:ti,ab,kw AND '1

hydroxy 4 methyl 3 penten 1 yl':ti,ab,kw AND

-:ti,ab,kw OR 'isoarnebin 4':ti,ab,kw OR '1,4

naphthalenedione,5,8 dihydroxy 2':ti,ab,kw) AND

'1 hydroxy 4 methyl 3 pentenyl':ti,ab,kw AND

-,:ti,ab,kw AND r:ti,ab,kw AND -:ti,ab,kw OR '1,4

naphthalenedione,5,8 dihydroxy 2':ti,ab,kw)

AND 1r:ti,ab,kw AND '1 hydroxy 4 methyl 3

pentenyl':ti,ab,kw AND -:ti,ab,kw OR 2-:ti,ab,kw)

AND 1r:ti,ab,kw AND '1 hydroxy 4 methyl 3

pentenyl':ti,ab,kw AND '5,8 dihydroxy 1,4

naphthoquinone':ti,ab,kw OR shikonin,:ti,ab,kw)

AND +:ti,ab,kw AND -isomer:ti,ab,kw OR

+:ti,ab,kw) AND -shikonin:ti,ab,kw OR r:ti,ab,kw)

AND -:ti,ab,kw AND +:ti,ab,kw AND

-shikonin:ti,ab,kw OR '5,8 dihydroxy 2':ti,ab,kw)

AND 1r:ti,ab,kw AND '1 hydroxy 4 methyl 3 penten

1 yl':ti,ab,kw AND '1,4

naphthalenedione':ti,ab,kw OR '1,4

naphthoquinone,5,8 dihydroxy 2':ti,ab,kw) AND '1

hydroxy 4 methyl 3 pentenyl':ti,ab,kw AND

-,:ti,ab,kw AND +:ti,ab,kw AND -:ti,ab,kw OR

+-:ti,ab,kw) AND -shikonin:ti,ab,kw OR '5,8

dihydroxy 2':ti,ab,kw) AND '1 hydroxy 4 methyl 3

pentenyl':ti,ab,kw AND '1,4

naphthalenedione':ti,ab,kw OR

acetylshikonin,:ti,ab,kw) AND +-:ti,ab,kw AND

-isomer:ti,ab,kw OR acetylshikonin,:ti,ab,kw) AND

s:ti,ab,kw AND -isomer:ti,ab,kw OR

acetylshikonin,:ti,ab,kw) AND r:ti,ab,kw AND

-isomer:ti,ab,kw OR 1r:ti,ab,kw) AND -1-:ti,ab,kw

AND '5,8 dihydroxy 1,4 dioxo 1,4 dihydro 2

naphthalenyl':ti,ab,kw AND

'-4-methyl-3-penten-1-yl acetate':ti,ab,kw OR

'arnebin i':ti,ab,kw OR 'dmask':ti,ab,kw OR

'beta, beta-dimethylacrylshikonin,':ti,ab,kw) AND

-:ti,ab,kw AND -isomer:ti,ab,kw OR 'beta,

beta-dimethylacrylshikonin,':ti,ab,kw) AND

+:ti,ab,kw AND -isomer:ti,ab,kw OR

'beta-hivs':ti,ab,kw OR 'shikonin analog

93-637':ti,ab,kw OR 'sa 93-637':ti,ab,kw

#5. ((('acetylshikonin':ti,ab,kw OR 1 17 Jun 2025

'beta,beta-dimethylacrylshikonin':ti,ab,kw OR

'isovalerylshikonin':ti,ab,kw OR

'beta-hydroxyisovalerylshikonin':ti,ab,kw OR

'deoxyshikonin':ti,ab,kw OR 'arnebinone':ti,ab,kw

OR 'alkannin':ti,ab,kw OR

'propionylshikonin':ti,ab,kw OR '2 methyl n

butyl':ti,ab,kw) AND shikonin:ti,ab,kw OR '5,8

dihydroxy 2':ti,ab,kw) AND '1 cinnamoyloxy 4

methylpent 3 enyl':ti,ab,kw AND '1,4

naphthoquinone':ti,ab,kw OR 1-:ti,ab,kw) AND '5,8

dihydro 1,4 dihydroxy 5,8 dioxo 2

naphthyl':ti,ab,kw AND '-4-methylpent-3-en-1-yl

cinnamate':ti,ab,kw OR

'beta,beta-dimethylacryloyl alkannin':ti,ab,kw OR

'beta-acetoxyisovalerylshikonin':ti,ab,kw

#4. 'arnebia*' 433 17 Jun 2025

#3. 'dextro alkannin'/exp OR 'dextro alkannin' OR 2,397 17 Jun 2025

'shikonin'/exp OR 'shikonin' OR 'shikonin

derivative'/exp OR 'shikonin derivative' OR

'acetylshikonin'/exp OR 'acetylshikonin' OR 'beta

beta dimethylacrylshikonin' OR 'beta beta

dimethylacrylshikonin' OR

'isovalerylshikonin'/exp OR 'isovalerylshikonin'

OR 'deoxyshikonin'/exp OR 'deoxyshikonin' OR

'arnebinone'/exp OR 'arnebinone' OR '(1 hydroxy 3

isohexenyl) naphthazarine'/exp OR '(1 hydroxy

3 isohexenyl) naphthazarine' OR '2 (1 hydroxy

4 methyl 3 pentenyl) 5, 8 dihydroxy 1, 4

naphtoquinone'/exp OR '2 (1 hydroxy 4 methyl 3

pentenyl) 5, 8 dihydroxy 1, 4 naphtoquinone' OR

'5, 8 dihydroxy 2 (1 hydroxy 4 methyl 3 pentenyl)

1, 4 naphthoquinone'/exp OR '5, 8 dihydroxy 2

(1 hydroxy 4 methyl 3 pentenyl) 1, 4

naphthoquinone' OR '5, 8 dihydroxy 6 (1 hydroxy 4

methyl 3 pentenyl) 1, 4 naphthoquinone'/exp OR

'5, 8 dihydroxy 6 (1 hydroxy 4 methyl 3

pentenyl) 1, 4 naphthoquinone' OR 'alkanna

red'/exp OR 'alkanna red' OR 'anchusa

acid'/exp OR 'anchusa acid' OR 'anchusin'/exp OR

'anchusin' OR 'ci 75530'/exp OR 'ci 75530' OR 'ci

natural red 20'/exp OR 'ci natural red 20' OR

'shikalkin'/exp OR 'shikalkin' OR 'alkannin'/exp

OR 'alkannin' OR 'propionylshikonin'/exp OR

'propionylshikonin' OR 'alkannin derivative'/exp

OR 'alkannin derivative'

#2. 'boraginaceae'/exp OR 'boraginaceae' OR 3,995 17 Jun 2025

'lithospermum'/exp OR 'lithospermum' OR 'arnebiae

radix'/exp OR 'arnebiae radix' OR 'arnebia

euchroma'/exp OR 'arnebia euchroma' OR 'arnebia

euchroma extract'/exp OR 'arnebia euchroma

extract' OR 'zicao'/exp OR 'zicao'

#1. 'eczema in infancy and childhood'/exp OR 'eczema 75,185 17 Jun 2025

in infancy and childhood' OR 'eczema

in infant and child'/exp OR 'eczema in

infant and child' OR 'eczema infantum'/exp OR

'eczema infantum' OR 'eczema, infantile'/exp OR

'eczema, infantile' OR 'infantile eczematous

skin'/exp OR 'infantile eczematous skin' OR

'infantile eczema'/exp OR 'infantile eczema' OR

'atopic constitutional neurodermatitis'/exp

OR 'atopic constitutional neurodermatitis'

OR 'atopic eczema'/exp OR 'atopic eczema' OR

'atopic neurodermatitis'/exp OR 'atopic

neurodermatitis' OR 'coca sulzberger

disease'/exp OR 'coca sulzberger disease' OR

'coca sulzberger syndrome'/exp OR 'coca

sulzberger syndrome' OR 'dermatitis, atopic'/exp

OR 'dermatitis, atopic' OR 'eczema atopica'/exp

OR 'eczema atopica' OR 'eczema endogenous'/exp OR

'eczema endogenous' OR 'endogenous eczema'/exp OR

'endogenous eczema' OR 'neurodermatitis

constitutionalis'/exp OR 'neurodermatitis

constitutionalis' OR 'neurodermatitis

disseminata'/exp OR 'neurodermatitis disseminata'

OR 'neurodermatitis, atopic constitutional'/exp

OR 'neurodermatitis, atopic constitutional' OR

'atopic dermatitis'/exp OR 'atopic dermatitis'

Material S4. Search strategy in Corchrane

ID Search Hits

#1 MeSH descriptor: [Dermatitis, Atopic] explode all trees 2350

#2 'Dermatitis, Atopic' OR Atopic Dermatitides OR Atopic Dermatitis OR 'Dermatitides, Atopic' OR 'Neurodermatitis, Atopic' OR Atopic Neurodermatitides OR Atopic Neurodermatitis OR 'Neurodermatitides, Atopic' OR 'Neurodermatitis, Disseminated' OR Disseminated Neurodermatitides OR Disseminated Neurodermatitis OR 'Neurodermatitides, Disseminated' OR 'Eczema, Atopic' OR Atopic Eczema OR 'Eczema, Infantile' OR Infantile Eczema 6405

#3 MeSH descriptor: [Lithospermum] explode all trees 4

#4 'Gromwell*' OR Lithosperm* OR 'Puccoons' OR 'Puccoon' 30

#5 MeSH descriptor: [Boraginaceae] explode all trees 46

#6 'Tournefortia*' OR 'Cynoglossum*' OR 'Anchusa*' OR 'Alkanna*' OR 'Onosma*' OR 'Ehretia*' OR 'Arnebia*' OR Zicao OR 'Boraginaceae' 65

#7 shikonin* OR 'Acetylshikonin' OR 'beta,beta-dimethylacrylshikonin' OR 'Isovalerylshikonin' OR 'beta-hydroxyisovalerylshikonin' OR 'Deoxyshikonin' OR 'Arnebinone' OR alkannin* OR 'Propionylshikonin' OR 'beta,beta-dimethylacryloyl alkannin' OR 'beta-acetoxyisovalerylshikonin' 'isoarnebin 4' OR 'arnebin I' OR 'DMASK' 94

#8 #3 OR #4 OR #5 OR #6 OR #7 216

#9 #1 OR #2 6405

#10 #8 AND #9 4

Material S5. Search strategy in ProQuest AND Google scholar

("Atopic Dermatitis" OR "Atopic Eczema") AND (Lithospermum OR Alkanna OR Arnebia OR Zicao OR Boraginaceae OR shikonin OR alkannin)

Results: 188 and 12

Material S6. Search strategy in CNKI

allintitle: Atopic Dermatitis Lithospermum OR Alkanna OR Arnebia OR Boraginaceae OR Zicao

Material S7. Search strategy in CNKI

(TKA=(特应性皮炎 OR 特应性湿疹 OR 特应性神经性皮炎 OR 播散性神经性皮炎 OR 婴儿湿疹 OR Atopic Dermatitis) AND (TKA=(紫草 OR 紫草属 OR 新疆紫草 OR 紫草油 OR 紫草 OR 紫草素 OR 茈草 OR 紫丹)

17

Material S8. Search strategy in CBM

1) ((((("紫草"[不加权:扩展]) OR "紫草属"[不加权:扩展]) OR "新疆紫草"[不加权:扩展]) OR "紫草油"[不加权:扩展]) OR "紫草膏"[不加权:扩展]) OR "紫草素"[不加权:扩展] 2562

2) "紫草"[常用字段:智能] OR "茈草"[常用字段:智能] OR "紫丹"[常用字段:智能] OR "紫草膏"[常用字段:智能] OR "紫草素"[常用字段:智能] OR "紫草油"[常用字段:智能] OR "新疆紫草"[常用字段] OR "软紫草属"[常用字段:智能] OR "滇紫草属"[常用字段:智能] 3987

3) "*紫草素"[常用字段:智能] 725 2023-08-12 13:36:49.0

4) "皮炎, 特应性"[不加权:扩展] 3356 2023-08-12 13:37:39.0

5) "婴儿湿疹"[常用字段:智能] OR "播散性神经性皮炎"[常用字段:智能] OR "特应性皮炎"[常用字段:智能] OR "特应性湿疹"[常用字段:智能] OR "特应性神经性皮炎"[常用字段:智能] OR "湿疹,"[常用字段:智能] AND "婴儿"[常用字段:智能] 4671 2023-08-12 13:39:21.0

6) (#3) OR (#2) OR (#1) 3987

7) (#5) OR (#4) 4671

8) (#7) AND (#6) 27

Material S9. Search strategy in WanFang

((题名或关键词=(特应性皮炎 OR 特应性湿疹 OR 特应性神经性皮炎 OR 播散性神经性皮炎 OR 婴儿湿疹 OR Atopic Dermatitis)) OR (摘要=(特应性皮炎 OR 特应性湿疹 OR 特应性神经性皮炎 OR 播散性神经性皮炎 OR 婴儿湿疹))) AND ((题名或关键词=(紫草 OR 紫草属 OR 新疆紫草 OR 紫草油 OR 紫草 OR 紫草素 OR 茈草 OR 紫丹)) OR (摘要=(紫草 OR 紫草属 OR 新疆紫草 OR 紫草油 OR 紫草 OR 紫草素 OR 茈草 OR 紫丹)))

29

Material S10. Search strategy in CQVIP

((R=(特应性皮炎 OR 特应性湿疹 OR 特应性神经性皮炎 OR 播散性神经性皮炎 OR 婴儿湿疹 OR Atopic Dermatitis)) OR (M=(特应性皮炎 OR 特应性湿疹 OR 特应性神经性皮炎 OR 播散性神经性皮炎 OR 婴儿湿疹))) AND ((R=(紫草 OR 紫草属 OR 新疆紫草 OR 紫草油 OR 紫草 OR 紫草素 OR 茈草 OR 紫丹)) OR (M=(紫草 OR 紫草属 OR 新疆紫草 OR 紫草油 OR 紫草 OR 紫草素 OR 茈草 OR 紫丹)))

Result:69

Material S11. FIGURE S1 Subgroup analysis
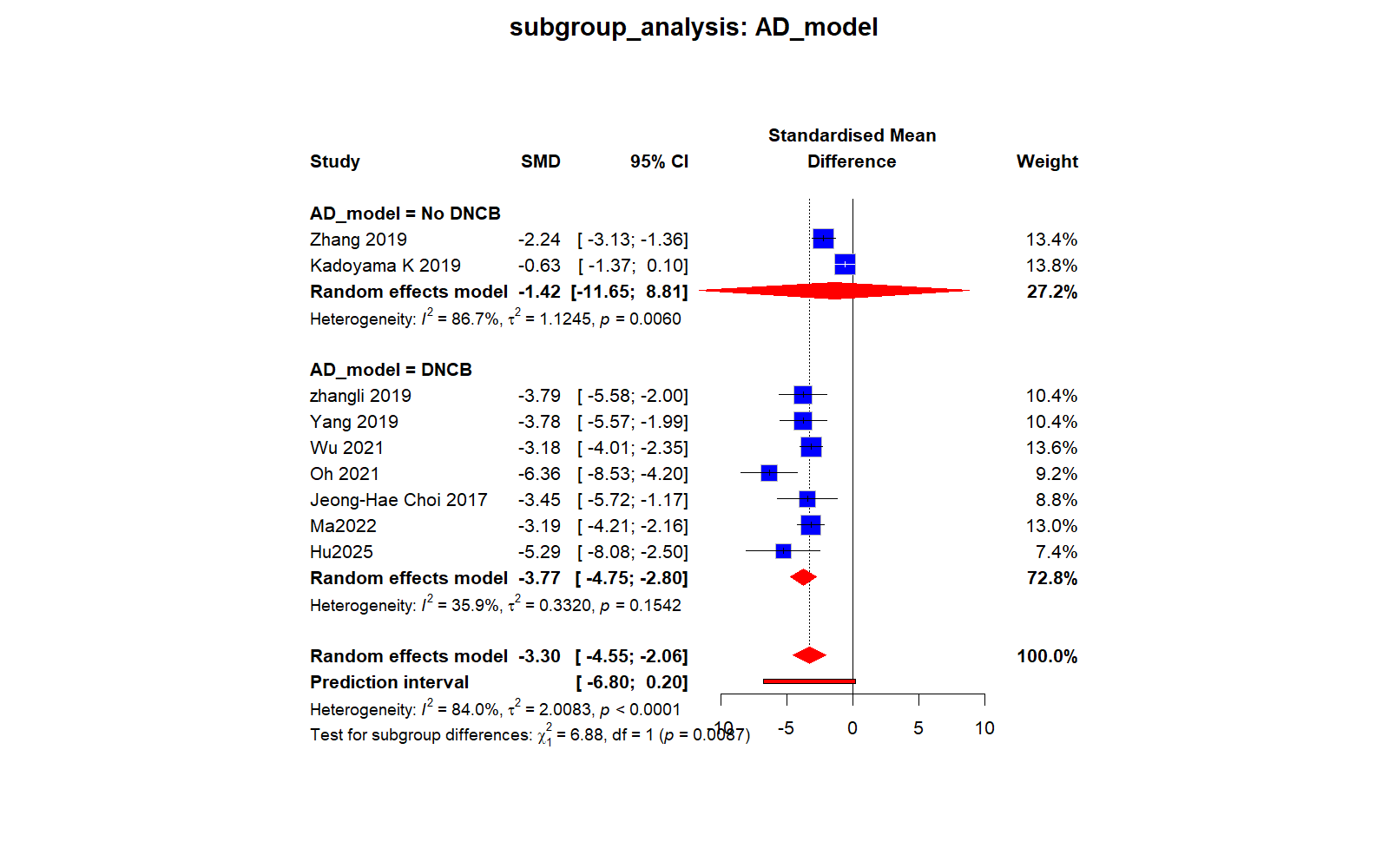


Fig 1 Meta-analysis of AD model subgroups


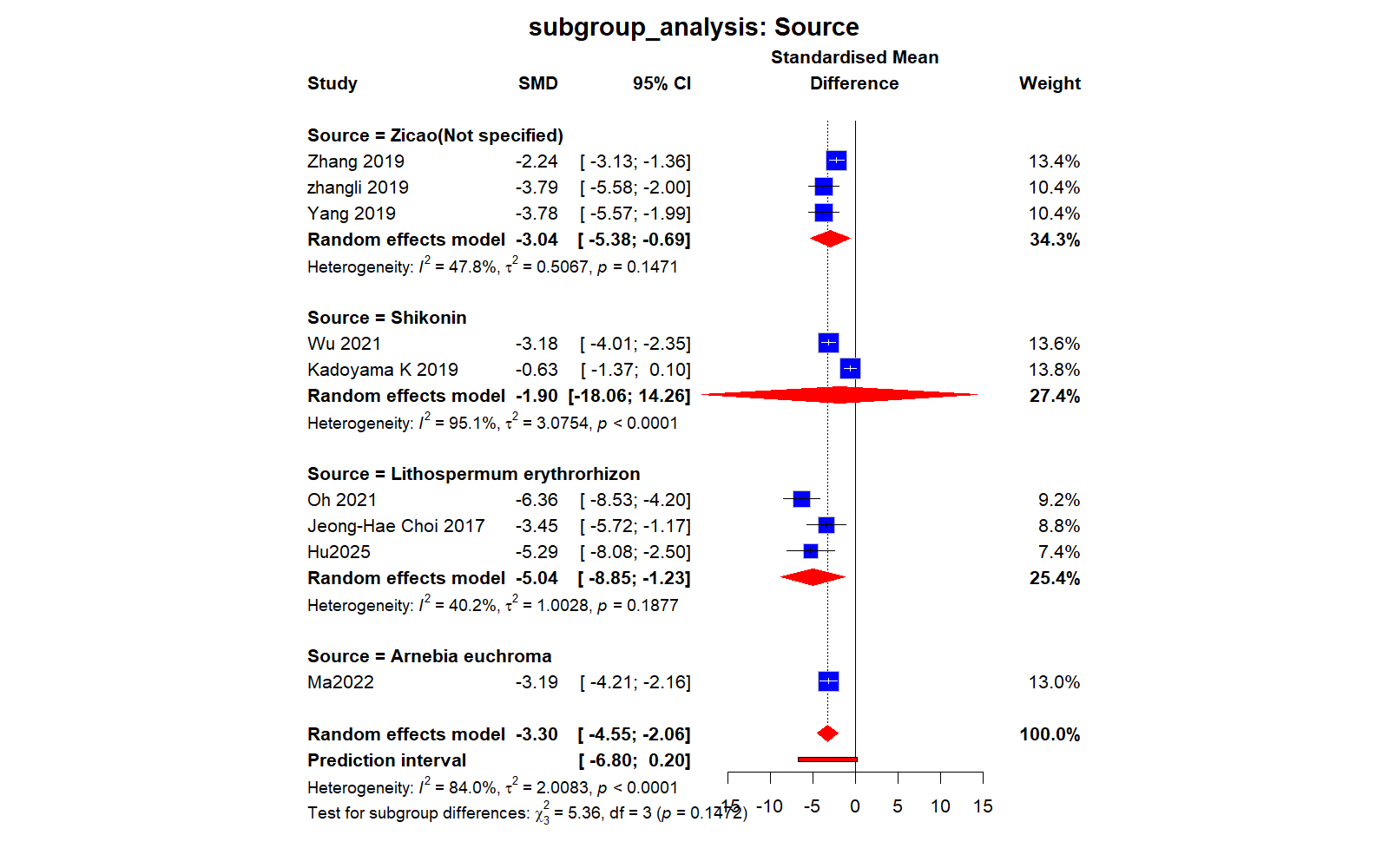


Fig 2 Meta-analysis of Source subgroups


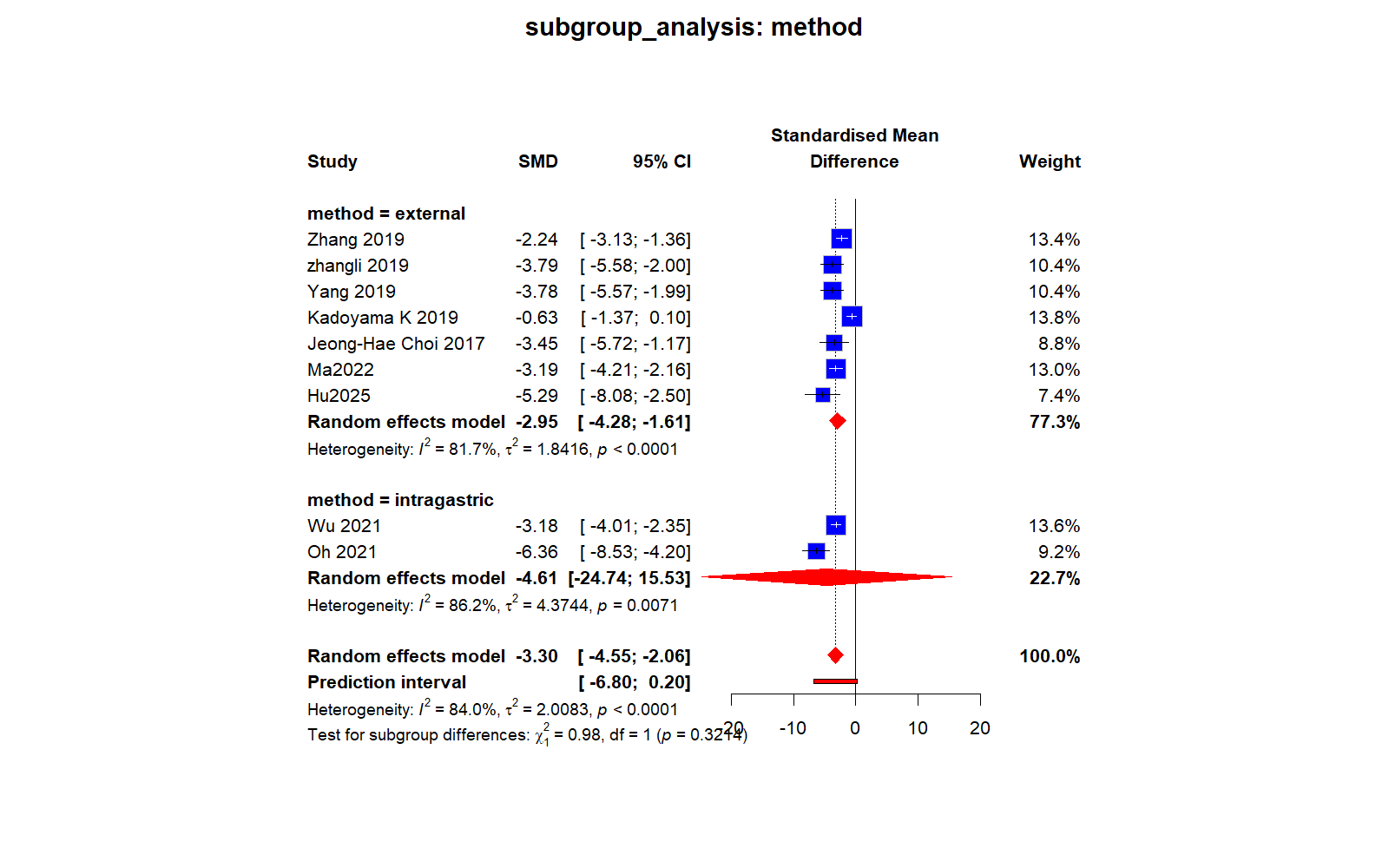


Fig 3 Meta-analysis of intervention method subgroups


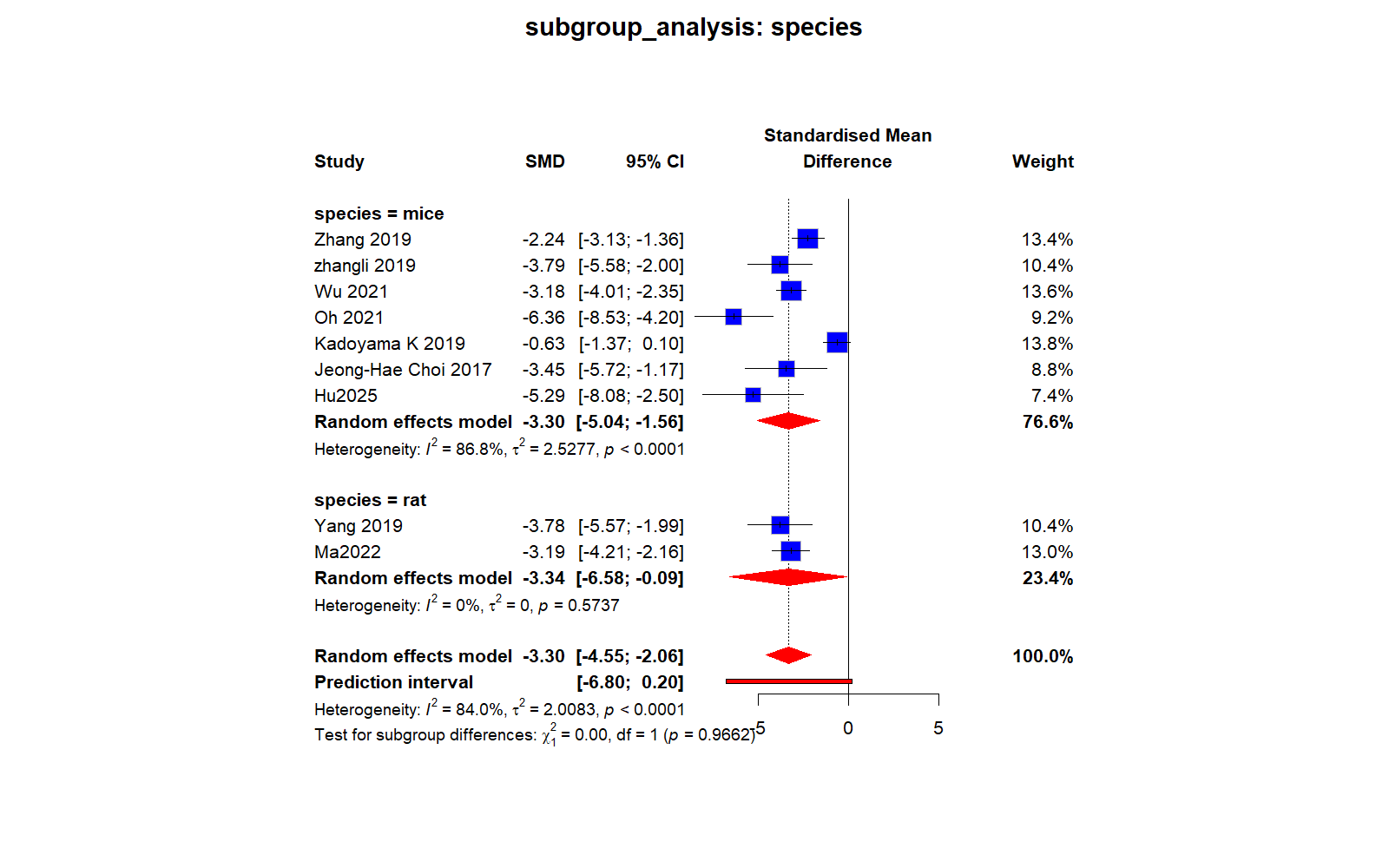


Fig 4 Meta-analysis of species subgroups

Fig 5 Meta-analysis of drug formulation subgroups


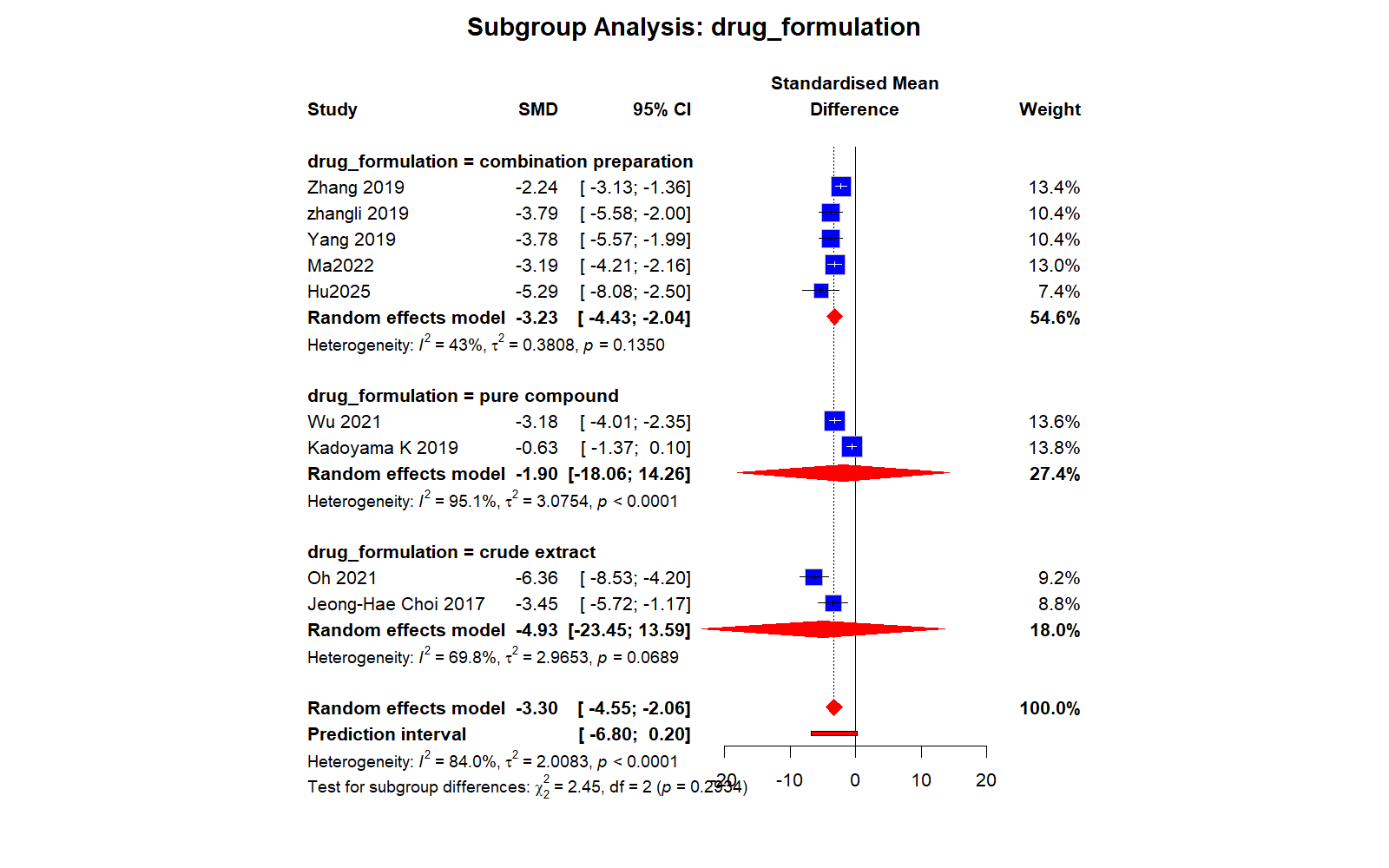


Material S12. FIGURE S2 influence analysis


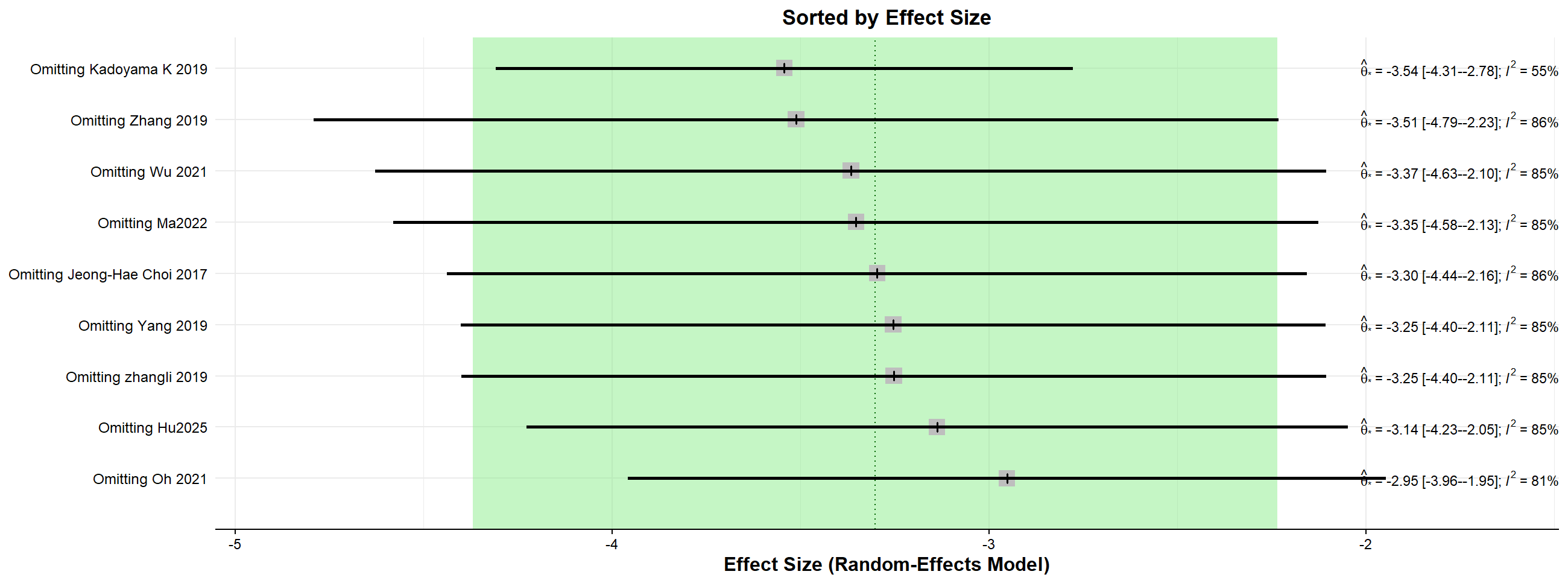


Fig 5 Leave-One-Out of Dermatitis severity on SMD


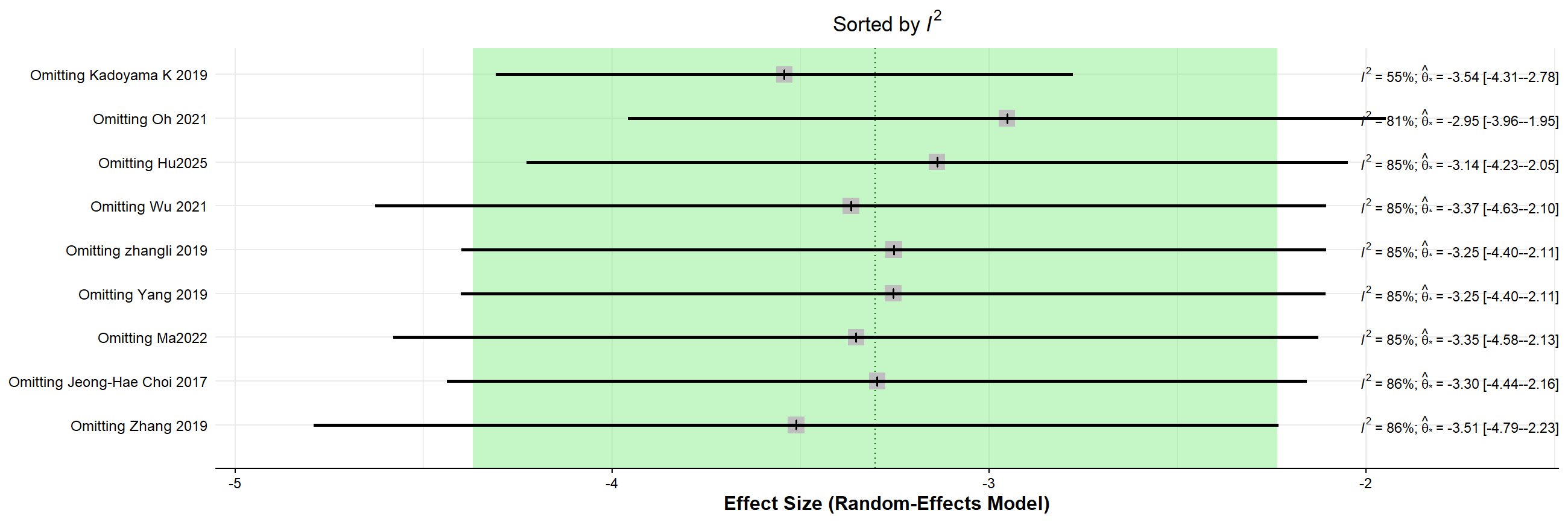


Fig 6 Leave-One-Out of Dermatitis severity on I^2^
